# Enabling an Inorganic-Rich Interface via Cationic Surfactant for High-Performance Lithium Metal Batteries

**DOI:** 10.1007/s40820-024-01364-x

**Published:** 2024-03-04

**Authors:** Zejun Sun, Jinlin Yang, Hongfei Xu, Chonglai Jiang, Yuxiang Niu, Xu Lian, Yuan Liu, Ruiqi Su, Dayu Liu, Yu Long, Meng Wang, Jingyu Mao, Haotian Yang, Baihua Cui, Yukun Xiao, Ganwen Chen, Qi Zhang, Zhenxiang Xing, Jisheng Pan, Gang Wu, Wei Chen

**Affiliations:** 1 Department of Chemistry, National University of Singapore, 3 Science Drive 3, Singapore, 117543 Singapore; 2 Agency for Science, Technology and Research (A*STAR), Institute of High-Performance Computing, 1 Fusionopolis Way, #16-16 Connexis, Singapore, 138632 Singapore; 3 Department of Physics, National University of Singapore, 2 Science Drive 3, Singapore, 117542 Singapore; 4 Joint School of National University of Singapore and Tianjin University, International Campus of Tianjin University, Binhai New City, Fuzhou, 350207 People’s Republic of China; 5 Agency for Science, Technology, and Research (A*STAR), Institute of Materials Research and Engineering, Innovis, 2 Fusionopolis Way, #08-03, Singapore, 138634 Singapore

**Keywords:** Cationic surfactant, Lithium nitrate additive, Solid–electrolyte interphase, Electric double layer, Lithium metal batteries

## Abstract

An anion-rich electric double layer (EDL) region is favorable for fabricating an inorganic-rich solid–electrolyte interphase (SEI) towards stable lithium metal anode in ester electrolyte. Herein, cetyltrimethylammonium bromide (CTAB), a cationic surfactant, is adopted to draw more anions into EDL by ionic interactions that shield the repelling force on anions during lithium plating. In situ electrochemical surface-enhanced Raman spectroscopy results combined with molecular dynamics simulations validate the enrichment of NO_3_^−^/FSI^−^ anions in the EDL region due to the positively charged CTA^+^. In-depth analysis of SEI structure by X-ray photoelectron spectroscopy and time-of-flight secondary ion mass spectrometry results confirmed the formation of the inorganic-rich SEI, which helps improve the kinetics of Li^+^ transfer, lower the charge transfer activation energy, and homogenize Li deposition. As a result, the Li||Li symmetric cell in the designed electrolyte displays a prolongated cycling time from 500 to 1300 h compared to that in the blank electrolyte at 0.5 mA cm^−2^ with a capacity of 1 mAh cm^−2^. Moreover, Li||LiFePO_4_ and Li||LiCoO_2_ with a high cathode mass loading of > 10 mg cm^−2^ can be stably cycled over 180 cycles.

**Supplementary Information:**

The online version contains supplementary material available at 10.1007/s40820-024-01364-x.

## Highlights

- Cetyltrimethylammonium cations can shield the repelling force on anions to attract more anions into electric double layer region during lithium plating process, facilitating the formation of inorganic-rich solid-state electrolyte interphase (SEI).
- An inorganic-rich (N/F-containing species) structure of SEI can be evidenced by the in-depth analysis.
- The cycling lifetime of Li||Li symmetric cell in the designed electrolyte can be extended from 500 to 1300 h. Moreover, full cells with a high cathode mass loading of >10 mg cm^-2^ can be stably cycled over 180 cycles.

**Supplementary Information:**

The online version contains supplementary material available at 10.1007/s40820-024-01364-x.

## Graphical abstract

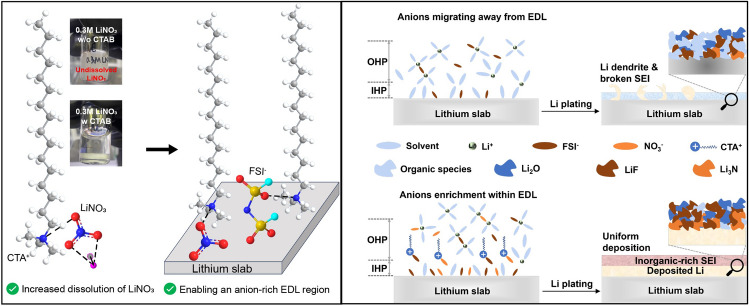

## Introduction

As modern society accelerates its transition from fossil fuel-driven energy to clean electricity, electrochemical storage systems inevitably emerge as energy intermediaries, encompassing electric vehicles, grid storage, and uninterruptible power supply (UPS) for data centers. With the evolution of lithium-ion batteries (LIBs) bottlenecked by the anode materials, lithium metal anodes (LMAs) hold great promise to supersede graphite anodes in the realization of high-energy–density batteries (> 400 Wh kg^−1^). LMAs are distinguished by two salient advantages, namely, substantial specific capacity (3860 mAh g^−1^) and low redox potential (−3.04 V vs. standard hydrogen electrode). However, their commercialization within batteries is impeded by two primary obstacles: the diminished coulombic efficiency (CE) and safety concerns arising from the growth of lithium dendrites.

Great efforts have been devoted to the field to enhance the performance of LMAs via optimizing the solid–electrolyte interphase (SEI), including developing (localized) high-concentration electrolytes [1, 2], introducing electrolyte additives [3, 4], designing novel Li salts or solvent [5, 6], and constructing artificial SEI [7, 8]. Among electrolyte additives, Li^+^ plating additives are less proposed than the film-forming additives as they regulate the Li^+^ plating pattern via a shielding mechanism or reduced diffusing barriers but do not get involved in the SEI formation [9, 10]. Cetyltrimethylammonium (CTA^+^), previous reported as Li^+^ plating regulator by lithiophobic repulsion, can suppress the growth of lithium dendrite, thus achieving long-term cycling of LMA [11]. Though it is a promising approach to realize uniform Li deposition by Li^+^ plating regulator, SEI with favorable species (e.g., Li_2_O, Li_3_N, and LiF) by film-forming additive is also important for durable LMA design. Furthermore, the composition and structure of the as-formed SEI are mainly dependent on the environment of electric double layer (EDL) on the LMA. To be specific, an EDL forms prior to the initial SEI formation, and thus the components inside EDL region are more likely to get reduced during the SEI formation process. However, previous reports solely focused on how CTA^+^ can uniform Li^+^ flux on the surface of LMA, the impact of CTA^+^ on EDL region was rarely investigated, let alone the enrichment of anions within EDL [12, 13]. Therefore, a new strategy is urgently required to combine these two additives into the electrolyte and the formation of anion-rich EDL region is beneficial for inorganic-rich SEI, which will enable an inorganic-rich SEI to facilitate the lateral growth of the deposited Li metal without Li dendrite growth.

Herein, cetyltrimethylammonium bromide (CTAB), a cationic surfactant, is adopted to promote the dissolution of favorable NO_3_^−^ anion and induce the formation of anion enrichment in the EDL region and therefore an inorganic-rich SEI. Specifically, the designed electrolyte (denoted as CNE-30) is prepared by dissolving 30 mM CTAB and 0.3 M LiNO_3_ in the blank electrolyte (1 M LiFSI in EC/DMC, denoted as BE). The unique anion enrichment within EDL region induced by CTA^+^ is well evidenced by in situ electrochemical surface-enhanced Raman spectroscopy (EC-SERS) and molecular dynamics (MD) simulations. The as-formed inorganic-rich SEI rendered by anion-rich EDL increases the average coulombic efficiency (ACE) to more than 99% and results in more than a twofold increase in the cycling lifetime of symmetric cells and full cells.

## Experimental Section

### Materials

Lithium bis(fluorosulfonyl)imide (LiFSI, > 99.9%), lithium difluoro(oxalato)borate (LiDFOB, > 99.9%), ethylene carbonate (EC, > 99.95%), and dimethyl carbonate (DMC, > 99.99%) were purchased from DoDo Chem. Lithium nitrate (LiNO_3_, > 99.99% metal basis) and cetyltrimethylammonium bromide (CTAB, > 99%) were purchased from Sigma-Aldrich. CTAB was vacuum-dried at 80 °C overnight and all solvents were further dried with 4 Å molecular sieve. Other Li salts were used without further purification. Lithium chips (600 μm) were purchased from China Energy Lithium Co., Ltd., China, and 50 or 100 μm Li was homemade via electric roller in the glovebox. Lithium iron phosphate (LiFePO_4_, LFP) and lithium cobalt oxide (LiCoO_2_, LCO) cathodes were purchased from Guangdong Canrd New Energy Technology Co., Ltd., China. Electrolytes were prepared in an Ar-filled glove box (Vigor Pte Ltd), in which both the content of O_2_ and H_2_O were lower than 0.5 ppm. BE electrolyte was prepared by dissolving 1 M LiFSI into EC/DMC 7:3 by volume. Additional 0.3 M LiNO_3_ with 30 mM CTAB in the BE electrolyte was denoted as CNE-30.

### Electrochemical Characterizations

2032-type coin-cell batteries were assembled in an Ar-filled glovebox (Vigor Pte Ltd). Li||Cu asymmetric cells were assembled with thick lithium foil (600 μm) as the anode (diameter, 14.3 mm), Cu foil as the counter electrode (diameter, 16 mm), and Celgard 3501 separator. The amount of electrolyte was about 60 μL. Li||LFP or Li||LCO full cells were assembled with thin lithium foil (100 μm) as the anode (diameter, 14 mm), LFP or LCO as the cathode (diameter, 12 mm), and an electrolyte of 20 μL. The active material loadings for LFP and LCO cathode were about 10.5 mg cm^−2^ and 10.3 mg cm^−2^, respectively. To avoid the corrosion of the stainless-steel positive cases by the electrolytes, Al-clad cathode cases were used for high-voltage battery tests [14]. All electrochemical batteries were performed on Neware battery testers. The electrochemical impedance spectroscopy (EIS) and cyclic voltammetry (CV) measurements were carried out on an AUTOLAB electrochemical workstation. CV profiles were obtained in a voltage window of 2.5 to 0 V or 1.0 to −0.2 V for the asymmetric Li||Cu cells. The temperature-dependent EIS spectra were collected in a symmetric cell of Li||Li with a frequency range of 10^5^–0.01 Hz and an AC voltage amplitude of 10 mV. Average coulombic efficiency (ACE) was measured as previously reported [15]. Specifically, 5 mAh cm^−2^ Li was deposited on Cu foil under 0.5 mA cm^−2^ for surface stabilization, after which the cell was charged to 0.5 V to fully strip the active Li. Then, another 5 mAh cm^−2^ Li was deposited on Cu foil as Li reservoir (*Q*_*T*_), and a fixed capacity of Li (*Q*_*C*_ = 1 mAh cm^−2^) was cycled between Cu and Li for 10/50 times, ending with stripping Li to 0.5 V (*Q*_*S*_). The ACE can be calculated by the following Eq. 1:

1 $${\text{ACE}} = \frac{{nQ_{C} + Q_{S} }}{{nQ_{C} + Q_{T} }}$$

where *n* is the cycling number; *Q*_*C*_, *Q*_*S*_, and *Q*_*T*_ are the fixed capacity of Li, stripping capacity of Li, and capacity of Li reservoir, respectively.

### Physical Characterizations

Scanning electron microscopy (SEM) images were obtained by a JEOL JSM-6701F FESEM. In situ optical microscopy (YM710R, Yuescope) was performed in a sealed two-electrode electrochemical cell. The cell was assembled with lithium chips as both working electrode and counter electrode and charged/discharged at 0.3 mA cm^−2^. Raman spectra were recorded at room temperature using a confocal WiTec Alpha 300R with laser excitation at 532 nm. The sample for X-ray photoelectron spectroscopy (XPS) was prepared by depositing 1 mAh cm^−2^ Li on Cu foil at a current density of 0.5 mA cm^−2^. XPS spectra were collected using an Omicron EA125 system with Al Kα (1486.7 eV) X-ray source. A home-built vacuum transfer chamber was implemented for transferring the samples from glovebox into XPS chamber to avoid degradation towards air. The sputtering was conducted using an Argon ion sputtering gun with operation energy of 1.0 keV at Argon pressure of 5.0 × 10^–5^ mbar. The sputtering rate is about 1.5 ± 0.5 nm min^−1^, depending on the composition and structure of the samples. Hence, the sputtering thickness in 20 min is about 30 ± 10 nm. ToF–SIMS 5 (ION-TOF GmbH, Germany) instrument was used for the ToF–SIMS depth profiling. The primary ion beam is Bi_1_^+^ (13.3 ns pulse width, 30 keV energy, 2 pA current), the primary analysis beam scan area is 100 μm × 100 μm. In the negative polarity ToF–SIMS depth profiling, the sputtering ion beam is Cs^+^ (2 keV energy, 90 nA current), Cs^+^ sputtered area 300 μm × 300 μm. The profile was acquired in interlaced mode. The data were acquired at a chamber pressure of 3.0 × 10^–8^ mbar.

### In Situ Surfaced-Enhanced Raman Spectroscopy (SERS)

A well-sealed three-electrode electrochemical cell with a transparent quartz window was selected to perform the in situ SERS. The cell was assembled with Cu mesh coated with Au nanoparticle as the working electrode, lithium chip as both the counter electrode and the reference electrode. During measurement, the voltage of the cell was controlled to drop from OCV to 0.2 V by electrochemical workstation (CHI 760e). Each Raman spectroscopy was recorded after the system reached equilibrium for 100 s at designated voltage.

### Molecular Dynamics (MD) Simulation

In this work, molecular dynamics (MD) simulations were conducted using the large-scale atomic/molecular massively parallel simulator (LAMMPS) [16, 17]. The bonded and nonbonded interactions were described using the condensed-phase optimized molecular potentials for atomistic simulation studies (COMPASS) force field [18, 19]. To account for long-range electrostatic interactions, the particle–particle particle–mesh (PPPM) method was employed [20, 21]. A time step of 1.6 fs was used consistently in all simulations, and periodic boundary conditions were applied in all directions to mimic an infinite system. To maintain a desired temperature of 298.15 K and pressure of 1 atm, the Nose–Hoover thermostat and barostat were utilized.

Two different electrolyte models were constructed to represent the CTAB-contained electrolyte and the blank electrolyte, following the experimental molar ratios. The CTAB-contained model consisted of 100 LiFSI, 1049 EC, 356 DMC, 3 CTAB, and 30 LiNO_3_ molecules, while the blank electrolyte comprised 100 LiFSI, 1049 EC, and 356 DMC molecules. To improve ensemble averaging, three distinct configurations were generated for each model.

After arranging the specific numbers of molecules in simulation boxes with dimensions of 41 × 41 × 120 Å^3^, a multistage equilibration protocol was applied to compress the system to the target density while relaxing internal stress [22]. The calculated density of the electrolyte, 1.290 ± 0.002 g cm^−3^, closely matched the experimental value of 1.328 g cm^−3^. Subsequently, radial distribution functions (RDFs) and distribution profiles of molecules were obtained from an NPT production stage of 10 ns.

Next, the equilibrated electrolyte models were sandwiched between two graphene sheets with different net charges to emulate batteries under different biases. The two graphene slabs represented the positive and negative electrodes, respectively. For simplicity, the interaction between the electrodes and the electrolyte was assumed to be purely nonbonded, and the electrodes were held fixed during the production stage. To eliminate the long-range electrostatic interaction between the mirrored slabs caused by periodic boundary conditions, a vacuum layer of 10 nm thickness was introduced perpendicular to the slabs. The sandwiched models underwent structure optimization and equilibration in the NPT ensemble for 30 ns to prepare them for subsequent production runs.

During the production stage, all equilibrated sandwiched models were maintained in the canonical ensemble (NVT) for 50 ns. RDFs and distribution profiles of molecules were sampled from the trajectories to identify the intermolecular interaction patterns.

## Results and Discussion

### CTAB as a Solubilizer and Solvation Structure

**Fig. 1:**
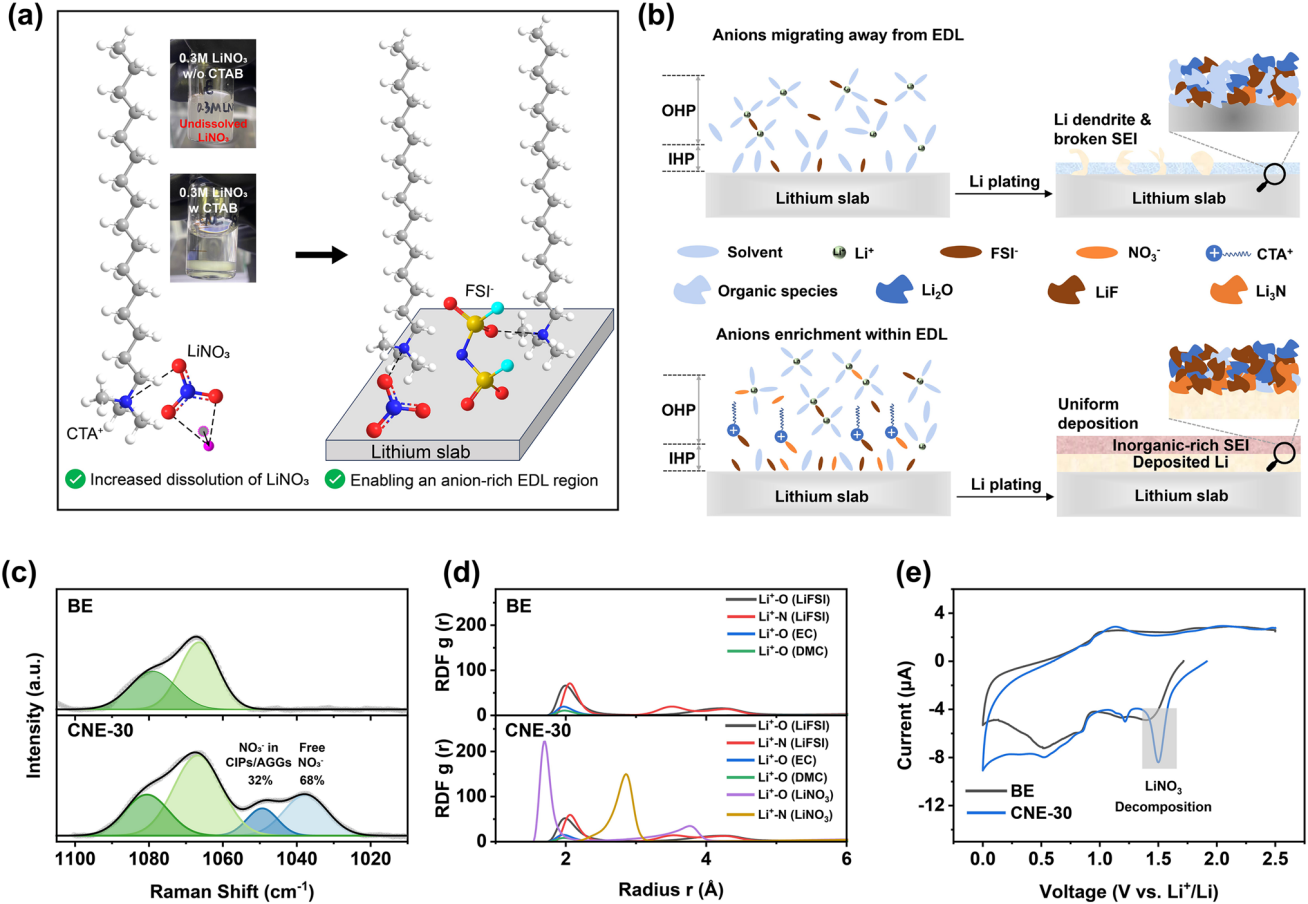
Characterization of the solvation structure and the schematic of the SEI formation. **a** Schematic of CTAB as a LiNO_3_ promoter as well as drawing anions into EDL with optical images of prepared electrolytes w and w/o CTAB. **b** Schematic of the proposed SEI formation process w and w/o the LiNO_3_ addition. **c** High-resolution Raman spectra of the BE and the CNE-30 electrolyte in the 1010–1105 cm^−1^ range. **d** The MD simulations of Li^+^-O and Li^+^-N radial distribution functions g(r) in the BE and the CNE-30 electrolyte. **e** CV curves of Li||Cu cells scanned at 0.1 mV s^−1^ in the voltage range of 0–2.5 V vs. Li^+^/Li

As shown in Fig. 1a, CTA^+^ can increase the solubility of LiNO_3_ by interacting with NO_3_^−^ thus pulling NO_3_^−^ away from Li^+^ (Fig. S1). At the same time, CTA^+^, due to its inherent electrochemical inertness and positive charge, maintains electrochemical stability during the Li deposition process and induces the formation of an anion-enriched EDL. As a result, a turbid liquid can be observed in the BE electrolyte with 0.3 M LiNO_3_, while a clear solution can be formed in the presence of CTAB. The ionic conductivity and transference number for the CNE-30 electrolyte are 12.2 mS cm^−1^ and 0.4, respectively, surpassing those of the BE electrolyte at 9.3 mS cm^−1^ and 0.22 (Fig. S2). The higher transference number for the CNE-30 electrolyte results from CTA^+^ attracting more anions from bulk electrolyte to EDL, thus immobilizing the anions and increasing the transference number. The SEI formed in the BE electrolyte and the CNE-30 electrolyte can be depicted through the schematic representation in Fig. 1b. To be specific, with the addition of solubilizing CTAB, the high-concentration (0.3 M) LiNO_3_ additive and FSI^−^ are preferentially reduced on the surface of LMA to establish a durable and inorganic-rich SEI, which can facilitate the fast Li^+^ transport and homogenize Li deposition. However, the SEI formed in the BE electrolyte is mainly originated from solvent reduction, inducing porous and organic-rich SEI with a poor ionic conductivity and uneven Li plating with serious Li dendrite formation issues.

The effect of high concentration of LiNO_3_ additive on the Li^+^-solvation structure was evaluated through Raman spectra. As shown in Fig. 1c, the peaks at 1037 and 1047 cm^−1^ correspond to the free NO_3_^−^ and the NO_3_^−^ in the contact ion pairs (CIPs)/aggregates (AGGs) states, respectively, indicating the effective participation of NO_3_^−^ in the Li^+^-solvation structure. Besides, the evolution of solvent and FSI^−^ anions in the Li^+^-solvation structure was also explored through fitting the characteristic peaks in the Raman spectra, demonstrating the increased proportion of FSI^−^ anions in CIPs/AGGs (Figs. S3 and S4). MD simulations were further conducted to evaluate the effect of LiNO_3_ on Li^+^-solvation structure in the bulk electrolyte (Fig. 1d). Through comparing the solvation sheath of the BE and the CNE-30 electrolyte, it can be found that the radial distribution functions of the CNE-30 electrolyte display the dominate peak of NO_3_^−^ (1.76 Å) in the first Li^+^-solvation shell (< 3 Å), indicating the strong interaction between Li^+^ and NO_3_^−^ [23]. The strongly coordinated NO_3_^−^ anions in the Li^+^-solvation and the lower LUMO of NO_3_^−^ than that of solvents will contribute to the preferential reduction of NO_3_^−^ before the decomposition of solvents, inducing the inorganic-rich SEI on the surface of LMAs.

For evaluating the SEI formation, the cyclic voltammetry (CV) profiles of the Li||Cu cells with the BE and the CNE-30 electrolyte were collected at 0.1 mV s^−1^ in the voltage range of 0–2.5 V. As shown in Fig. 1e, the reduction peaks at around 1.5 and 1.2 V can be attributed to the decompositions of LiNO_3_ and LiFSI, respectively, which can be obviously observed in the CNE-30 electrolyte. The preferential reduction of anions induces an inorganic-rich and dense SEI in the CNE-30 electrolyte, mitigating the further reduction of electrolyte in the second-cycle CV profile (Fig. S5).

### Exploration of EDL Evolution

**Fig. 2:**
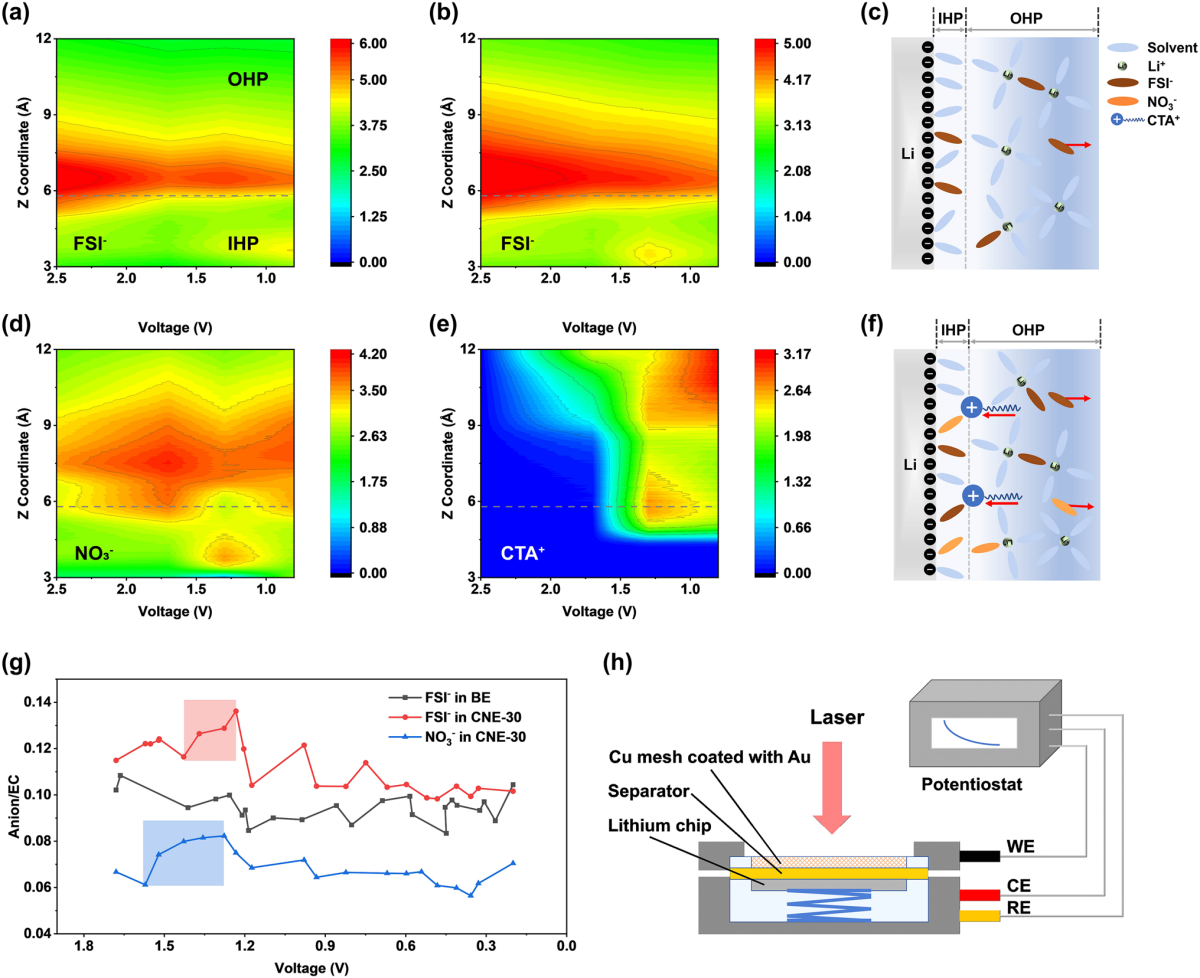
Exploration of EDL evolution by MD simulations and in situ EC-SERS during the first SEI formation. Intensities of FSI^−^/EC ratios in **a** the BE electrolyte, **b** the CNE-30 electrolyte and **c** NO_3_^−^/EC ratio, **d** CTA^+^ in the CNE-30 electrolyte as a function of distance (Å) from graphene electrode under different applied voltages (V). Schematic of EDL model during the first SEI formation in **e** the BE and **f** the CNE-30 electrolyte. **g** Calculated anion/EC ratios from 1.7 to 0.2 V according to Raman peaking fitting. **h** Schematic of in situ EC-SERS cell configuration: Cu mesh coated with Au nanoparticles as the working electrode, lithium chip as both the counter electrode and the reference electrode

In addition to the Li^+^-solvation structure in the bulk electrolyte, the evolution of the component distribution in the EDL region has significant influence on the initial SEI formation, which will be further explored by MD simulations and in situ EC-SERS under various voltages. As shown in Fig. 2a, b and S6, FSI^−^/EC ratios in the BE and the CNE-30 electrolyte were plotted as a function of distance (Å) from graphene electrode under different applied voltages (V). Typically, the Inner Helmholtz plane (IHP) is thinner than 1 nm within EDL region, which can only hold single ion/molecular layer on electrode surface [13]. In this work, the thickness of IHP is determined to be 5.8 Å according to a peak position of solvent nearest to graphene electrode (Fig. S7). Along with voltage drop from 2.5 to 0.8 V, the component distribution in EDL region is dramatically varied, greatly influencing the subsequent SEI formation. Generally, with the increase in density of electron on graphene electrode, anions will be repulsed away from the IHP while the cations attracted to the IHP. In the BE electrolyte (Fig. 2a), the FSI^−^/EC peak displays attenuated intensity in the IHP region with the decrease in voltages, indicating that FSI^−^ anions are migrating away from the IHP. However, in the CNE-30 electrolyte (Fig. 2b, d), significant enrichment of the FSI^−^ and NO_3_^−^ can be observed in the IHP region when the voltage drops below 1.5 V. Besides, the decrease in the FSI^−^/EC intensity is obviously mitigated compared to that in the BE electrolyte. Interestingly, as shown in Fig. 2e, CTA^+^ tends to accumulate in the OHP region and the boundary between the OHP and the IHP when the voltage drops from 2.5 to 1.6 V, exhibiting a pattern of enrichment analogous to that of anions in the CNE-30 electrolyte. The accumulation of CTA^+^ in the EDL region can be attributed to the applied electric field force, which expulses anions but attracts cations with the decrease in voltages. Due to the steric hindrance, CTA^+^ cannot enter the IHP but stay at the outer boundary of IHP. Consequently, the electrostatic interaction between CTA^+^ and anions (i.e., NO_3_^−^, FSI^−^) dramatically relieves the migration of anions away from the EDL and induces the enrichment of anions in the IHP at specific voltage. One snapshot taken from MD simulations can also verify the enrichment of both NO_3_^−^/FSI^−^ and CTA^+^ near LMA (Fig. S8). Based on the preceding simulation results, the illustrative diagram of anion enrichment within EDL region is presented in Fig. 2c, f**.** To be specific, the force exerted by the electric field, pushing anions away from the anode, is shielded by CTA^+^ in the CNE-30 electrolyte, resulting in a higher retention of anions within the EDL region. Therefore, NO_3_^−^ and FSI^−^ undergo preferential reduction to render a dense and inorganic-rich SEI on the surface of LMA. In contrast, driven by electric field force, anions move away from the anode in the BE electrolyte, leading to a greater occupancy of solvent molecules within the EDL region. Consequently, the SEI formed in the BE electrolyte is mainly derived from solvent reduction.

Empirical data, in conjunction with MD simulations, can be used to substantiate the EDL evolution and anion enrichment in EDL region. Hence, to better identify the electrolyte species on electrode surface experimentally, in situ EC-SERS was applied to detect the high-resolution spectra during the electrochemical process. As shown in Fig. 2h, laser was focused on a copper mesh coated with gold nanoparticles to detect the vibration spectroscopy during SEI formation. During the measurement, in situ EC-SERS cell was connected to an electrochemical workstation and controlled to discharge from open circuit voltage (OCV) to 0.2 V as not to form Li–Au alloy on electrode. As shown in Fig. S9, representative Raman spectra under the specific voltages in the BE and the CNE-30 electrolyte were obtained and well fitted to distinguish electrolyte species with different states. Specifically, the Raman peaks at 731 and 738 cm^−1^ can be attributed to free FSI^−^ and FSI^−^ in CIPs/AGGs, respectively, and both peaks were selected for ratio calculation [24]. Similarly, the Raman peaks at 889 and 899 cm^−1^ arise from free EC and solvated EC; 1037 and 1047 cm^−1^ from free NO_3_^−^ and NO_3_^−^ in CIPs/AGGs, respectively [25]. FSI^−^/EC and NO_3_^−^/EC ratios were obtained to compare the relative intensity of anions within EDL region during the SEI formation process. As shown in Fig. 2g, in the BE electrolyte, the FSI^−^/EC ratio does not show obvious enrichment during voltage drop, while in the CNE-30 electrolyte, the FSI^−^/EC ratio shows a significant increase from 1.4 to 1.2 V and gradual decrease in following voltages. Moreover, the NO_3_^−^/EC ratio exhibits a pronounced increase from 1.6 to 1.3 V, subsequently diminishing under successive voltages. Considering the abovementioned results, we validate that the anions (FSI^−^ and NO_3_^−^) can accumulate on the electrode surface under specific voltages with help of CTA^+^, a conclusion consistent with prior theoretical simulation outcomes and the proposed SEI formation mechanism.

### In-depth Analysis of SEI Structure

**Fig. 3:**
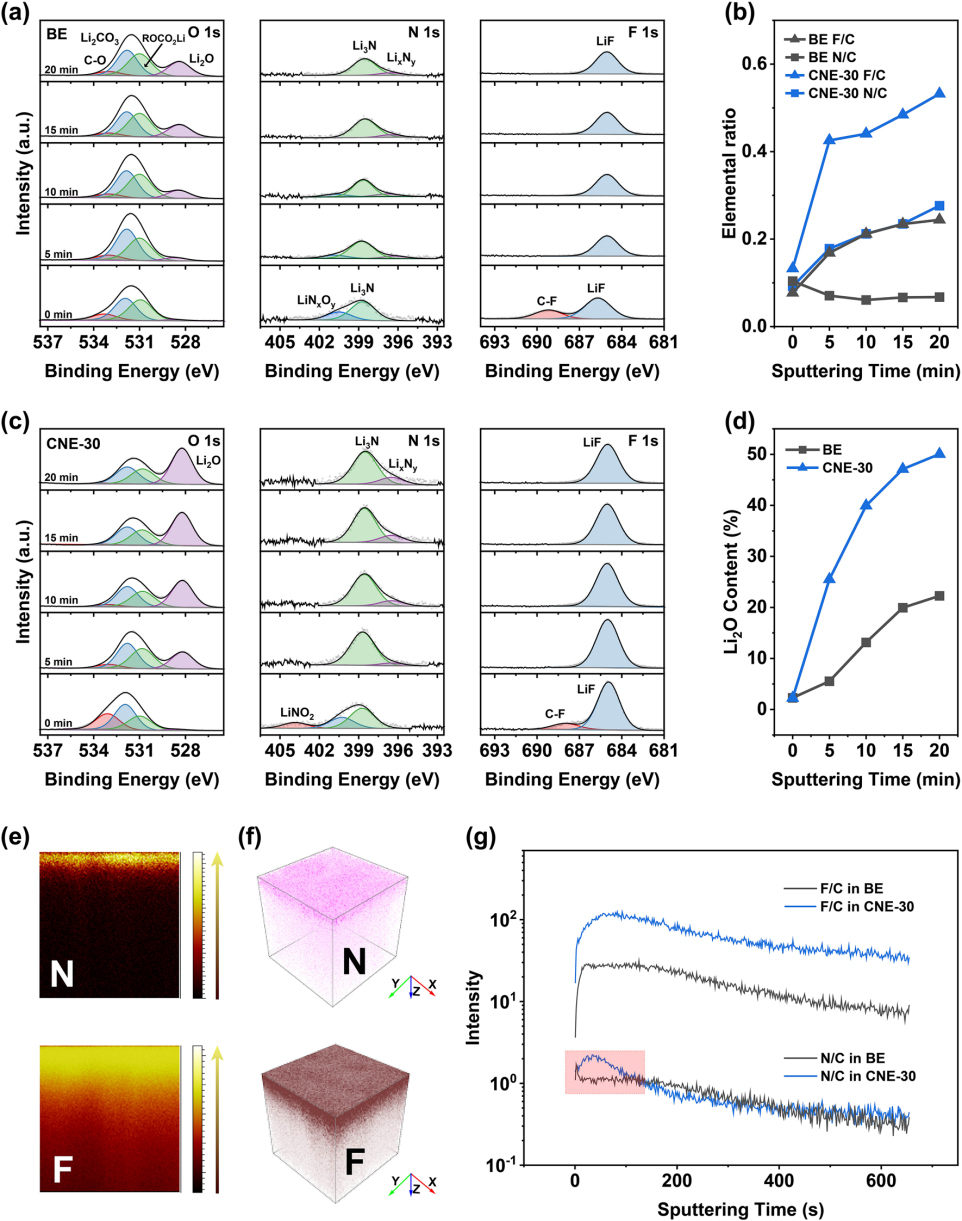
In-depth analysis of chemical structure of SEI by XPS and ToF–SIMS. XPS spectra of O 1s, N 1s, and F 1s for SEI formed in **a** the BE and **c** the CNE-30 electrolyte and the corresponding fitted curves. The detections were conducted on electrochemically deposited Li on Cu foil after five cycles, and the Li surface was sputtered by Ar^+^ for 5, 10, 15, and 20 min, respectively. **b** Elemental ratios of F/C and N/C in the BE and the CNE-30 electrolyte after the different sputtering times. **d** Li_2_O content (%) in the BE and the CNE-30 electrolyte. The distribution of N and F formed in the CNE-30 electrolyte in **e** cross section and **f** 3D reconstructions via ToF–SIMS with a detection area of 100 μm × 100 μm. **g** Elemental ratios of N/C and F/C in the BE and the CNE-30 electrolyte via ToF–SIMS

The differences of the Li^+^-solvation structure and EDL environment in the BE and the CNE-30 electrolyte are bound to induce variations in the structure of the SEI, including chemical compositions and elemental distributions. Herein, the chemical structure of the as-formed SEI in different electrolytes was examined by XPS with subsequent Ar^+^ sputtering for 5, 10, 15, and 20 min, respectively. The sample was prepared by depositing Li of 1 mAh cm^−2^ on Cu foil after five cycles under 0.5 mA cm^−2^ and then transferred in the argon atmosphere without any exposure to air or moisture. In the N 1s spectra, the decompositions of LiFSI and LiNO_3_ additive can both contribute to the N-containing components in the SEI (Fig. 3a, c). Before sputtering, Li_3_N and LiN_*x*_O_*y*_ dominate on the surface of the as-formed SEI in both electrolytes. Moreover, LiNO_2_, the incomplete decomposition product from LiNO_3_, can be found on the as-formed SEI in the CNE-30 electrolyte, indicating the sustained migration of LiNO_3_ additive from the bulk electrolyte to the Li surface. With the increase in sputtering time, the LiN_*x*_O_*y*_ disappears and Li_3_N/Li_*x*_N_*y*_ become the dominant compositions in the as-formed SEI. Similarly, lithium fluoride (LiF), the efficient decomposition product of LiFSI, dominates in the F 1*s* spectra after sputtering (Fig. 3a, c). Although C-F (689 eV) exists on the surface of the as-formed SEI in both electrolytes, distinct differences lie in the content of C-F which is 29.8% in the BE and 6.6% in the CNE-30 electrolyte, indicating a more complete reduction of LiFSI in the CNE-30 electrolyte (Fig. S10).

In the electrolyte system, F comes solely from LiFSI, N comes from LiFSI/LiNO_3_, and C comes from carbonate solvent. Therefore, the ratios of F/C and N/C under various sputtering time can be normalized to reflect the content of inorganic components in SEI formed in the BE and the CNE-30 electrolyte. As shown in Figs. 3b and S11, the elemental ratios of F/C and N/C increase with the sputtering time in both electrolytes. However, for the spectra collected at each sputtering time, the calculated F/C and N/C elemental ratios display much larger values in the CNE-30 electrolyte than those in the BE electrolyte, indicating the inorganic-dominant SEI structure in the CNE-30 electrolyte. The higher F/C and N/C elemental ratios in the CNE-30 electrolyte can be attributed to the anion-rich Li^+^-solvation structure and the anion-dominant EDL environment. Besides, the presence of LiNO_3_ additive can promote the complete reduction of LiFSI near Li surface [26], also contributing to the higher F/C ratios in the CNE-30 electrolyte. Noteworthy is that the ratio of N/C in the inner layer (close to the LMA) in the CNE-30 can be even three times larger than the one in the BE electrolyte. Besides, the O 1s spectra of the SEI in the CNE-30 electrolyte also display much higher content of Li_2_O than those in the BE electrolyte, especially in the inner layer of the as-formed SEI, (Fig. 3a, c), reflecting the preferential reduction of anions (i.e., NO_3_^−^, FSI^−^) on LMA in the CNE-30 electrolyte. With continuous Ar^+^ sputtering, the content of Li_2_O located at 528.6 eV unremittingly increases, ending up being the dominant component in the spectrum. After 20 min of Ar^+^ sputtering, the content of Li_2_O can rise to 20% and 50% in the BE and the CNE-30 electrolyte, respectively (Fig. 3d).

The chemical structure of SEI and elemental distribution were further examined by time-of-flight secondary ion mass spectrometry (ToF–SIMS). A square of 100 $\times$ 100 μm Li was selected as detection area under Ga-ion beam in negative mode. Distinct signals of N and F are found on the top layer of SEI formed in the CNE-30 electrolyte, in accordance with the XPS results that LiFSI/LiNO_3_ decomposes completely on Li surface (Fig. 3e, f). Moreover, the ratios of F/C are always higher in the CNE-30 electrolyte than those in the BE electrolyte with the increase in sputtering time (Figs. 3g and S12). It is worth noting that N-containing species in the CNE-30 electrolyte are concentrated on the surface of SEI and show the richest distribution near the surface. Such uniform distribution of N/F-containing spices across SEI facilitates the fast and homogeneous transportation of Li^+^ and conduces to the rapid two-dimensional (2D) diffusion of Li^+^ along the inorganic SEI/LMA interface with high interfacial energy.

### Electrochemical Characterization of Asymmetric and Symmetric Cells

**Fig. 4:**
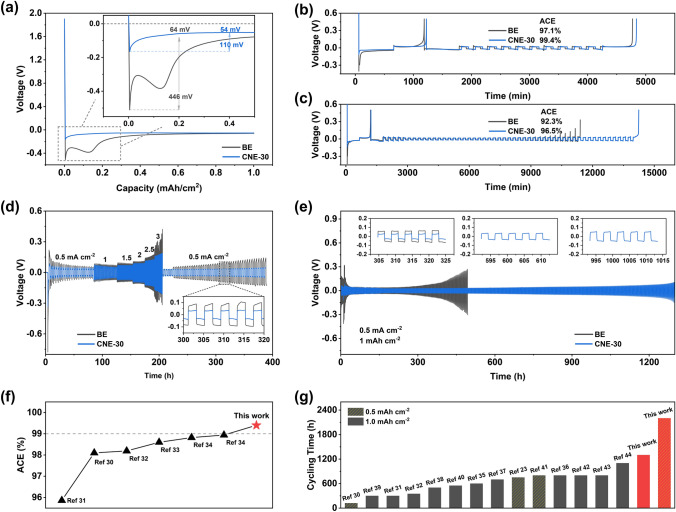
Average CE measurement in asymmetric cells and cycling performance of symmetric cells with the BE and the CNE-30 electrolyte. **a** Galvanostatic discharge curves in Li||Cu half cells under 0.5 mA cm^−2^/1 mAh cm^−2^ during the 1st cycle and the inset indicates the nucleation overpotential (${\eta }_{1}$) and mass-transfer overpotential (${\eta }_{2}$). Average CE measurement after **b** 10 cycles and **c** 50 cycles. **d** Rate performance of the Li||Li cells under 0.5/1/1.5/2/2.5/3 mA cm^−2^ and a fixed capacity of 1 mAh cm^−2^. **e** Comparison of long-term cycling of the Li||Li cells under 0.5 mA cm^−2^/1 mAh cm^−2^ with insets showing magnified overpotential at 300 h/600 h/1000 h, respectively. Performance comparison between our work and recent advances in **f** ACEs and **g** symmetric cells

Before coulombic efficiency (CE) measurements in Li||Cu cells, five cycles of 0–2.5 V discharging/charging under 0.05 mA cm^−2^ were performed to establish a stable interface on Cu foil. After the activation, the 1st plating curve (Fig. 4a) can reveal the nucleation overpotential (${\eta }_{1}$) and mass-transfer overpotential (${\eta }_{2}$). To be specific, the ${\eta }_{1}$ and ${\eta }_{2}$ values in the BE electrolyte are 446 and 64 mV, respectively. Comparatively, the Li||Cu cell displays much smaller ${\eta }_{1}$ (110 mV) and ${\eta }_{2}$ (54 mV) values in the CNE-30 electrolyte, indicating a smaller nucleation barrier and mass-transfer resistance in the CNE-30 electrolyte [27]. It is worth mentioning that the voltage dip of the Li||Cu cell using the BE electrolyte appears twice, but the counterpart of the cell using the CNE-30 electrolyte appears only once. The first voltage dip of Li||Cu cell using the BE electrolyte is mainly attributed to the initial Li nucleation process which requires overpotential of 446 mV to form Li seeds on Cu foil. Then, the overpotential begins to decrease. However, during the subsequent Li deposition on Cu foil, the SEI rendered from carbonate electrolyte is incompatible with the Li and breaks down, exposing fresh Li to electrolyte. The second voltage dip is mainly from the exposure of fresh nucleation sites caused by the organic-dominated unstable SEI. Decomposition of electrolyte and thickening of the SEI will further increase the overpotential. When the newly generated nucleation sites are fully covered by the Li deposition, the voltage will again decrease to a plateau (${\eta }_{2}$) [28, 29]. For Li||Cu cell using the CNE-30 electrolyte, a similar explanation is applied for the first voltage dip during Li deposition. However, the SEI rendered with carbonate electrolyte and LiNO_3_ additive that is mechanically durable will not break down during Li deposition. As a result, no second voltage dip appears on the voltage-current curve. The CE was further evaluated by long-term cycling of Li||Cu cells under 0.5 mA cm^−2^/1 mAh cm^−2^. As shown in Fig. S13, the CE of the BE rapidly decayed after the 50th cycle, while that of the CNE-30 maintains stably over 120 cycles with an average coulombic efficiency (ACE) of 95.35% in the first 100 cycles. Moreover, a method proposed by Zhang et al. was used for accurate determination of ACE, which can eliminate the effect of substrate conditions and assess the reversibility of LMA in the given electrolyte [15]. As a result, the cells with the CNE-30 electrolyte display high ACE values, i.e., 99.4% for 10 cycles and 96.5% for 50 cycles (Fig. 4b, c**)**, which are higher than most of the reported values (Fig. 4f) [30–34]. Yet for the BE electrolyte, the ACE value is less than 99% after 10 cycles and the cell gets short circuit before 50 cycles, unsatisfactory for practical cell operation.

Apart from the high ACE values of the CNE-30 electrolyte measured in the asymmetric cells, symmetric cells were also fabricated to demonstrate the superior interfacial stability of SEI formed in the CNE-30 under the cycling condition of 0.5 mA cm^−2^/1 mAh cm^−2^. As shown in Fig. 4e, Li||Li cells with the CNE-30 electrolyte can be stably cycled for 1300 h with overpotentials of ~ 50 mV, while Li||Li cells with the BE electrolyte suffer larger overpotential after 500 h. It is worth noting that during the early stage of cycling (~ 15 h), the overpotential of Li||Li cells with the BE electrolyte rises to 300 mV, indicating large interfacial resistance. However, the overpotential of Li||Li cells with the CNE-30 electrolyte can quickly stabilize at ~ 50 mV, which can be attributed to the stable SEI and the reduced interfacial resistance, resulting from the inorganic decomposition product of LiNO_3_ additive. Also, the long-term cycling performance of Li||Li cells is compared among carbonate electrolytes with different concentrations of CTAB (Fig. S14). Moreover, the long-term cycling lifetime of Li||Li cells with the CNE-30 electrolyte outperforms most of the reported results in the studies (Fig. 4g) [23, 30–32, 35–44]. When cycled under a moderate condition of 0.5 mA cm^−2^/0.5 mAh cm^−2^, the cycling time of the Li||Li cells with the CNE-30 electrolyte can even be extended to more than 2200 h, while those with the BE electrolyte fail after 1200 h (Fig. S15). Cycling lifetime under higher current density is also compared between the BE and the CNE-30 electrolyte (Fig. S16). Furthermore, different current densities (0.5–3 mA cm^−2^) were applied to Li||Li cells to evaluate the rate performance (Fig. 4d). The overpotential of Li||Li cells with the CNE-30 electrolyte remains stable even at 3 mA cm^−2^ and quickly returned to ~ 35 mV when the current density changes from 3 to 0.5 mA cm^−2^. In contrast, the Li||Li cells with the BE electrolyte get short circuit at 3 mA cm^−2^ and display a larger overpotential of 100 mV when the current density returns to 0.5 mA cm^−2^. As indicated in the abovementioned results, the addition of LiNO_3_ additive is beneficial for reducing nucleation/mass-transfer overpotentials, improving ACE, and enhancing rate performance as well as long-term cycling in symmetric cells which can be attributed to the inorganic-rich SEI established by preferential reduction of anions (i.e., FSI^−^ and NO_3_^−^) in the CNE-30 electrolyte.

### Interfacial Kinetics and Li Deposition Morphology

**Fig. 5:**
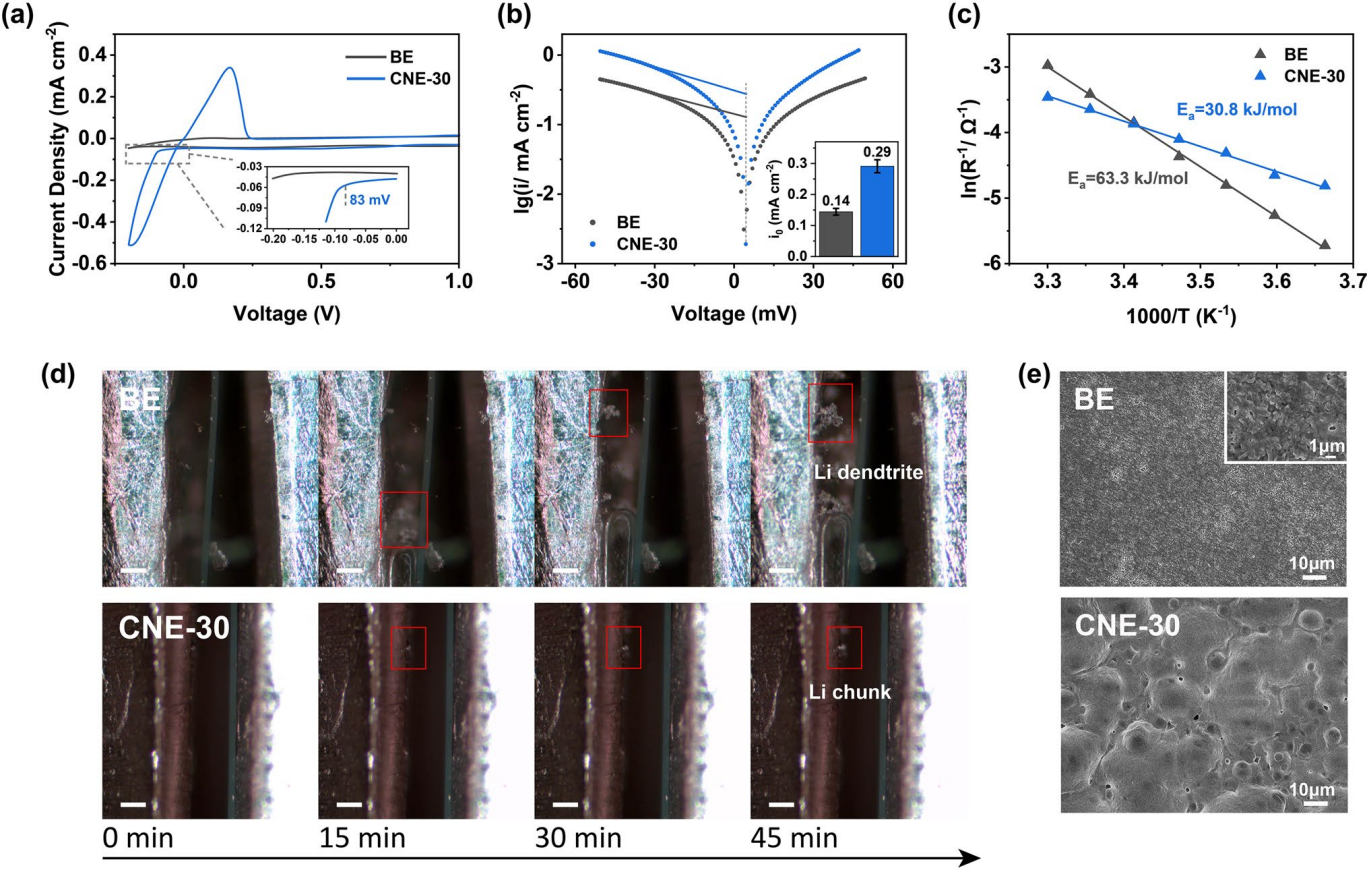
The kinetics of Li^+^ transfer at Li/electrolyte interface. **a** CV curves of the BE and the CNE-30 electrolyte under 0.1 mV s^−1^ in the voltage range of −0.2 to 2.5 V vs. Li^+^/Li. **b** Tafel plots obtained by the Li||Li cells of the BE and the CNE-30 electrolyte with inset showing the exchange current densities (*i*_0_). **c** Charge transfer activation energies *E*_*a*_ calculated using Arrhenius equation. **d** In situ optical images of Li deposition in the BE and the CNE-30 electrolyte (scale bars, 100 μm). **e** SEM images of Li deposition morphology

CV was performed on the Li||Cu cells with the BE and the CNE-30 electrolyte to examine the Li reversibility and plating overpotential at a scan rate of 0.1 mVs^−1^ in the voltage range of −0.2 to 2.5 V vs. Li^+^/Li. As indicated in Fig. 5a, during the plating process, the Li||Cu cell with the CNE-30 electrolyte shows a lower overpotential of 83 mV for initial deposition than that of the BE (150 mV). Besides, the stripping current peak for the CNE-30 (0.34 mA cm^−2^) is much higher than that of the BE (< 0.01 mA cm^−2^). Exchange current density (i_0_), collected from Tafel measurement, describes the charge transfer capability and kinetics of Li^+^ transfer at LMA/electrolyte interface. As shown in Fig. 5b, the exchange current density of the CNE-30 (0.29 mA cm^−2^) is nearly twice that of the BE (0.14 mA cm^−2^), revealing the improved Li plating/stripping kinetics. Each measurement was repeated four times and the error bar was shown in the inset. The superior Li^+^ transfer kinetics can be attributed to the inorganic-dominant and uniform SEI formed in the CNE-30 electrolyte, which promotes the fast and homogeneous Li^+^ diffusion through SEI.

Furthermore, electrochemical impedance spectroscopy (EIS) analysis was performed on the Li||Li cells with the BE and the CNE-30 electrolyte under a series of temperatures ranging from 273 to 303 K (Fig. S17 and Table S2). The interfacial resistance (lnR^−1^) was plotted against temperature (1000/*T*) and fitted according to the Arrhenius Eq. 2 [45]:

2 $$\frac{1}{{R}_{{\text{SEI}}}}={A}_{0}{e}^{-{E}_{a}/RT}$$

where *A*_0_, *E*_*a*_, and *R* represent a pre-exponential constant, the activation energy, and the gas constant, respectively. As indicated in Fig. 5c, a distinct difference lies in the activation energy of Li^+^ transfer through SEI (*E*_*a*_), where *E*_*a*_ reduces from 63.3 kJ mol^−1^ (BE) to 30.8 kJ mol^−1^ (CNE-30). Such a sharp drop in barrier of Li^+^ transfer through SEI can be ascribed to the higher content of highly Li^+^ conductive species (e.g., Li_3_N, LiF, and Li_2_O) in the SEI formed by the CNE-30. It should be noted that *R*_SEI_ has dynamic values instead of static ones with different temperatures and cycling numbers. As shown in Fig. S18 and Table S3, the values of R_SEI_ decrease gradually with cycling. To be specific, the interfacial resistance of the cells in the CNE-30 (153.6 Ω) is much lower than that of the cells in the BE electrolyte (1010.2 Ω) before cycling. The lower interfacial resistance in the CNE-30 electrolyte may be caused by primary reduction of LiNO_3_ additive by LMA spontaneously. SEI embedded with reduction products of LiNO_3_ has a higher Li^+^ conductivity, thus exhibiting lower interfacial resistance and smaller overpotential in symmetric cells.

The ionic conductivity of SEI greatly dictates the deposition morphology of lithium metal [46]. To be more specific, the higher the ionic conductivity, the larger the size of the deposited Li. As shown in Figs. 5d and S19, the pattern of lithium growth and stripping in the BE and the CNE-30 electrolyte was observed through in situ optical microscopy in which symmetric cells were assembled and cycled under 0.3 mA cm^−2^. It can be seen that after 45 min of Li plating on LMA, obvious dendrite appears in the BE electrolyte while chunky Li instead of Li dendrite can be clearly observed in the CNE-30 electrolyte. This difference can be attributed to the different SEI formed on Li electrode. LiF/Li_3_N-rich SEI formed in the CNE-30 electrode regulates the uniform Li^+^ during the plating process, thus no Li dendrite appearing on surface. Beyond macroscopic deposition morphology, SEM images can further illustrate the different deposition morphologies from a microscopic perspective. As can be seen in Fig. 5e, the deposited Li in the BE electrolyte is porous with a diameter of less than 1 μm, while the one in the CNE-30 is dense with diameter more than 10 μm. Porous Li with high surface area is expected to induce severe side reaction with electrolyte, contributing to the low CE value in the BE electrolyte.

### Electrochemical Performance of Full Cells

**Fig. 6:**
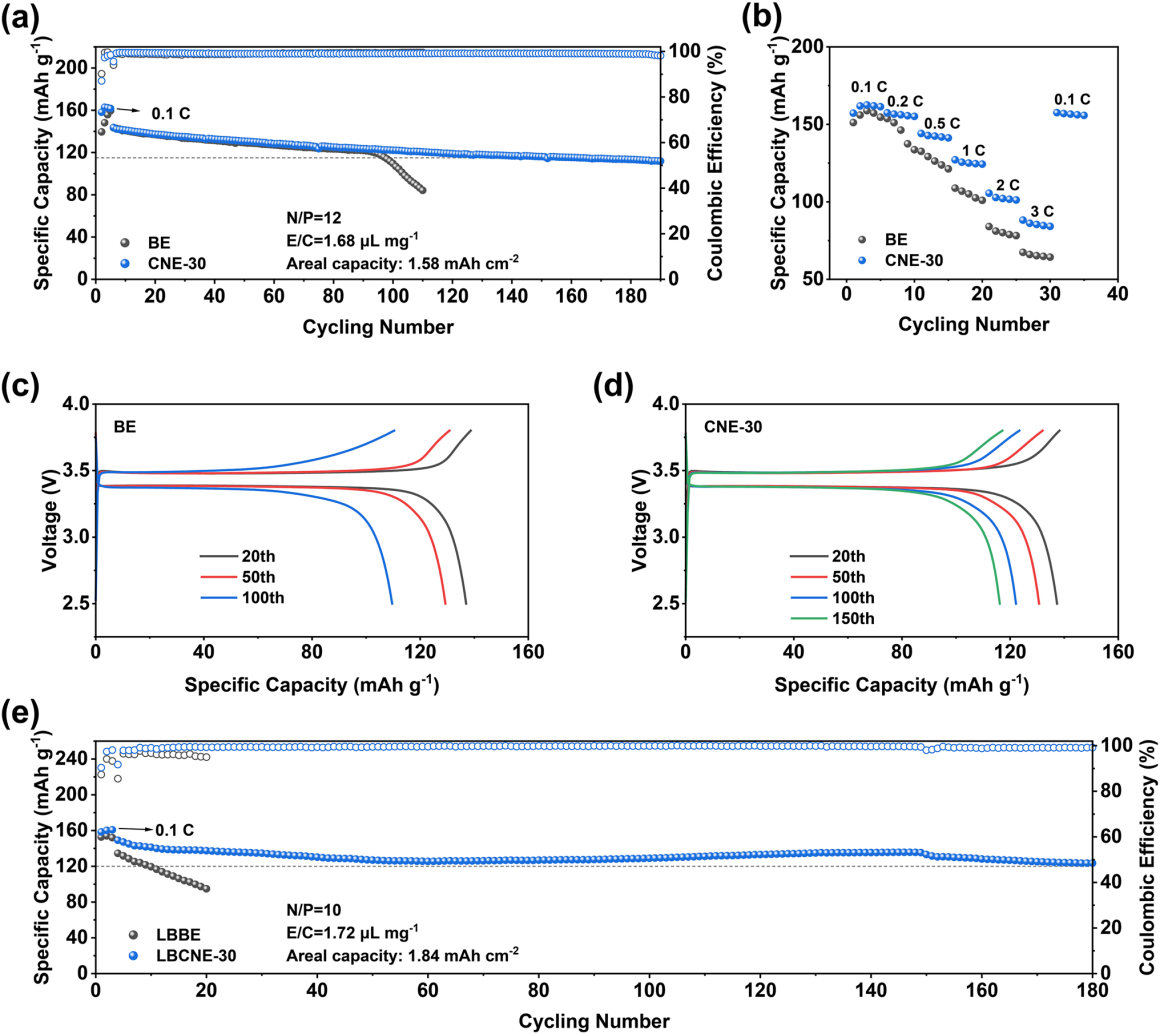
Full cell performance in the BE and the CNE-30 electrolyte. **a** Long-term cycling performance of the Li||LiFePO_4_ full cell with the BE and the CNE-30 in an electrolyte of 1.68 μL mg^−1^ with a N/P ratio of 12 and cathode loading of 10.5 mg cm^−2^ under 0.5 °C. **b** Rate performance of the Li||LiFePO_4_ full cell under 0.1/0.2/0.5/1/2/3 C with the BE and the CNE-30 electrolyte. Capacity–voltage curves during the 20th/50th/100th cycle with **c** the BE and **d** the CNE-30 electrolyte. **e** Long-term cycling performance of the Li||LiCoO_2_ full cells in an electrolyte of 1.72 μL mg^−1^ with an N/P ratio of 10 and cathode loading of about 10.3 mg cm.^−2^ under 0.5 C (charging)/1.0 C (discharging)

Though a high concentration of LiNO_3_ additive (0.3 M) was successfully introduced into carbonate electrolyte by CTAB, it cannot be efficiently decomposed on cathode surface within operation voltage since LiNO_3_ got reduced below 1.5 V. Wang et al. demonstrated that a LiF-rich cathode electrolyte interphase (CEI) can be formed by reduction of anions at 1.7 V (vs. Li^+^/Li) instead of an organic-rich CEI, which can elongate the cycling lifetime of the Li||LiCoO_2_ full cells [47]. This unique strategy can also be extended to the full cells of the Li||LiFePO_4_ to promote the LiNO_3_ decomposition on cathode side to form Li_3_N/Li_2_O-rich CEI. Before cycling under 0.5 °C, an activation process of charging to 3.8 V and discharging to 1.45 V followed with a potentiostatic discharging was repeated three times for a stable SEI/CEI. Then, the Li||LiFePO_4_ full cells were cycled under a practical condition: an electrolyte of 1.68 μL mg^−1^ with an N/P ratio of 12 and a high cathode mass loading of 10.5 mg cm^−2^ (Fig. 6a). The Li||LiFePO_4_ full cells with the BE electrolyte show fast capacity decay with 80% capacity retention after 100 cycles. In contrast, a prolonged cycling lifetime of the Li||LiFePO_4_ full cells with the CNE-30 electrolyte can be achieved with 80% capacity retention after 180 cycles, showing 80% increase compared to that of the Li||LiFeO_4_ full cells of the BE electrolyte. The capacity–voltage curves during 20th/50th/100th cycle can be found in Fig. 6c, d. The capacity of the Li||LiFePO_4_ full cell with the BE electrolyte drops by 15.5% from the 50th to 100th cycle, while that of the Li||LiFePO_4_ with the CNE-30 electrolyte only displays a neglectable loss (1.2%). At the same time, the superior rate capability of the Li||LiFePO_4_ full cells with the CNE-30 electrolyte is demonstrated in Fig. 6b through rate test, ranging from 0.1 to 3 C. Under high-rate conditions of 2 C and 3 C, the Li||LiFePO_4_ full cell with the CNE-30 electrolyte exhibits high capacities of 106 and 88 mAh g^-1^, respectively, while that with the BE electrolyte shows 84 and 67 mAh g^-1^, respectively. Such improved long-term cycling and rate performance can be contributed to LiF/Li_3_N/Li_2_O-rich SEI and CEI rendered by LiNO_3_.

Furthermore, the compatibility with high-voltage cathode was demonstrated by pairing LMA with lithium cobalt oxide cathode (LiCoO_2_). The intrinsic instability of solvent and Li salts at high-voltage (> 4.2 V) due to oxidation and corrosion can be mitigated by adding small amount of cathode additive, LiDFOB [34]. Therefore, the BE and the CNE-30 electrolyte were upgraded by 0.4 M LiDFOB to be the LBBE and the LBCNE-30 electrolyte for high-voltage (4.35 V) full cells. As shown in Fig. 6e, the cycling lifetime of the Li||LiCoO_2_ full cell with the LBCNE-30 gets extended to 180 cycles with 80% capacity retention, but the full cell with the LBBE suffers from fast capacity fading only after 10 cycles. The short cycling lifetime of the Li||LiCoO_2_ full cell with the LBBE can be attributed to severe side reactions at cathode interface when charged to 4.35 V. In contrast, the Li||LiCoO_2_ with the LBCNE-30 electrolyte only undergoes neglectable capacity loss (2.7%) from the 50th to 180th cycle (Fig. S20), demonstrating the improved oxidation stability of the electrolyte with the help of the high-concentration LiNO_3_ additive (0.3 M). Moreover, the rate performance of the full cells using both electrolytes is compared under different rates (Fig. S21). Despite the same amount of cathode additive dissolved in electrolyte, NO_3_^−^ can further improve the interfacial stability by altering the EDL region during charging process thus achieving better cycling stability [48]. The disparity in Li deposition morphology (Fig. S22) between the LBBE and the LBCNE-30 electrolyte together with various oxidation stabilities collectively explains the substantial performance differences in high-voltage full cells.

## Conclusions

CTAB has been demonstrated to attract more anions NO_3_^−^/FSI^−^ into the IHP within EDL region. The preferential decomposition of anions (e.g., NO_3_^−^, FSI^−^) within the EDL leads to a uniform and durable SEI dominated by inorganic substances (e.g., LiF, Li_3_N, and Li_2_O), ensuring fast interfacial kinetics and uniform Li deposition. In the rationally designed electrolyte, a significantly prolongated cycling lifetime from 500 to 1300 h of the Li||Li symmetric cell can be achieved under a cycling condition of 0.5 mA cm^−2^/1 mAh cm^−2^. Moreover, the Li||LiFePO_4_ and Li||LiCoO_2_ with a high cathode mass loading of > 10 mg cm^−2^ can retain 80% capacity after 180 cycles. This work not only introduces an efficient solubilizer (CTAB) to increase the solubility of LiNO_3_ in ester electrolyte, but also offers an in-depth understanding of the effect of CTAB on the well-maintained anion-rich EDL environment, which can disclose the design guideline of interfacial regulation towards inorganic-rich SEI for high-performance batteries.

## Supplementary Information

Below is the link to the electronic supplementary material.

Supplementary file1 (PDF 2084 KB)
