# Supplementary material for: Enabling an Inorganic-Rich Interface via Cationic Surfactant for High-Performance Lithium Metal Batteries
Source: Nanomicro Lett. 2024 Mar 4;16:141. doi: 10.1007/s40820-024-01364-x (PMC10912072; doi:10.1007/s40820-024-01364-x)
Supplement: Supplementary file 1 — Supplementary file1 (PDF 2084 KB) [file 40820_2024_1364_MOESM1_ESM.pdf]

Supporting Information for

Enabling an Inorganic-Rich Interface via Cationic Surfactant for High- Performance Lithium Metal Batteries

Zejun Sun [1,#], Jinlin Yang [1,#,*], Hongfei Xu [1], Chonglai Jiang [1,4], Yuxiang Niu [1], Xu Lian [1], Yuan Liu [1], Ruiqi Su[1], Dayu Liu[1], Yu Long [1,4], Meng Wang [1,4], Jingyu Mao [3], Haotian Yang [1,4], Baihua Cui [1,4], Yukun Xiao [1,4], Ganwen Chen [1,4], Qi Zhang [1], Zhenxiang Xing [5], Jisheng Pan [5], Gang Wu [2,*], Wei Chen [1,3,4,*]

[1] Department of Chemistry, National University of Singapore, 3 Science Drive 3, 117543, Singapore

[2] Institute of High-Performance Computing, Agency for Science, Technology and Research (A*STAR), 1 Fusionopolis Way, #16-16 Connexis, Singapore 138632, Singapore

[3] Department of Physics, National University of Singapore, 2 Science Drive 3, 117542, Singapore

[4] Joint School of National University of Singapore and Tianjin University, International Campus of Tianjin University, Binhai New City, Fuzhou, 350207, P. R. China

[5] Institute of Materials Research and Engineering, Agency for Science, Technology, and Research (A*STAR), 2 Fusionopolis Way, Innovis, #08-03, 138634, Singapore

[#] Zejun Sun and Jinlin Yang contributed equally to this work.

*Corresponding authors. E-mail: yjlchem@nus.edu.sg (Jinlin Yang), wug@ihpc.a-star.edu.sg (Gang Wu), phycw@nus.edu.sg (Wei Chen)

Supplementary Figures and Table

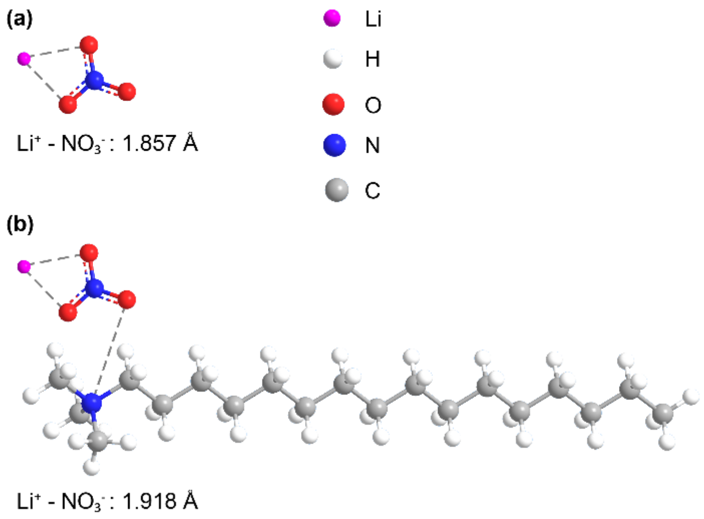

Fig. S1 Calculated distance of (a) Li^+–NO_3^- and (b) Li^+– NO_3^-: CTA^+

Notes: The distance between Li^+– NO_3^- increases from 1.857 to 1.918 Å with the addition of CTA^+. The coulombic interaction between CTA^+ and NO_3^- leads to an increased separation between Li^+ and NO_3^-, contributing to an efficient dissolution of $LiNO_3$ in the ester electrolyte.

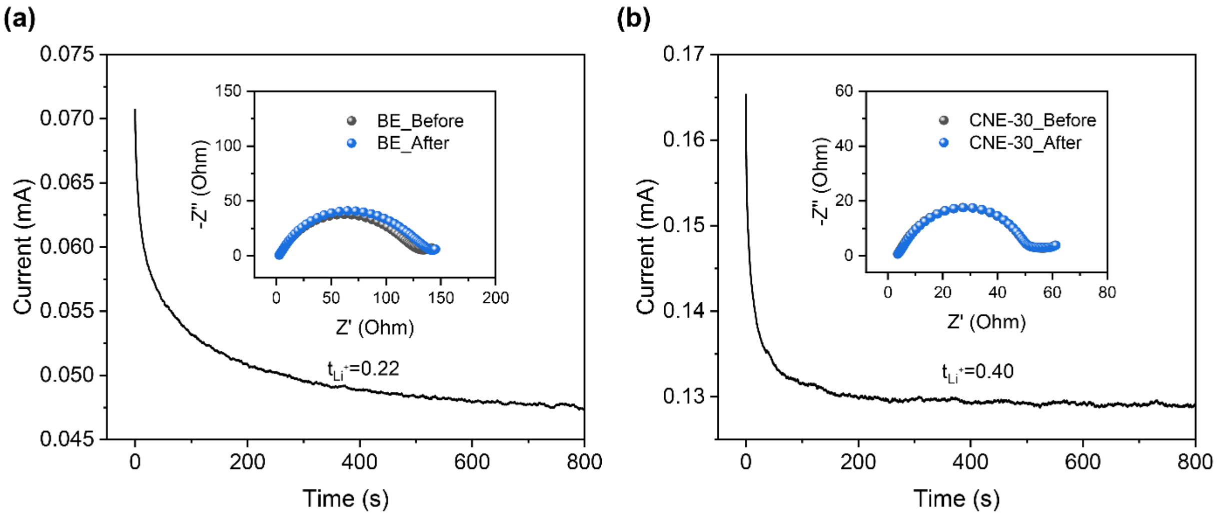

Fig. S2 Li$^+$ transference numbers of the (**a**) BE and (**b**) CNE-30 electrolyte

Notes: EIS measurements of symmetric cells using the BE and CNE-30 electrolyte were conducted before and after being applied polarization of 10 mV. The Li$^+$ transference number can be calculated via the Bruce and Vincent method using following Eq. S1:

$$t_{Li^+} = \frac{I_S(V-I_0R_0)}{I_0(V-I_SR_S)} \qquad \text{(S1)}$$

where t_{Li+} is the Li$^+$ transference number, V is the applied polarization voltage, I_0 and I_s are the initial and stabilized current, and R_0 and R_s are the initial and stabilized interfacial resistance.

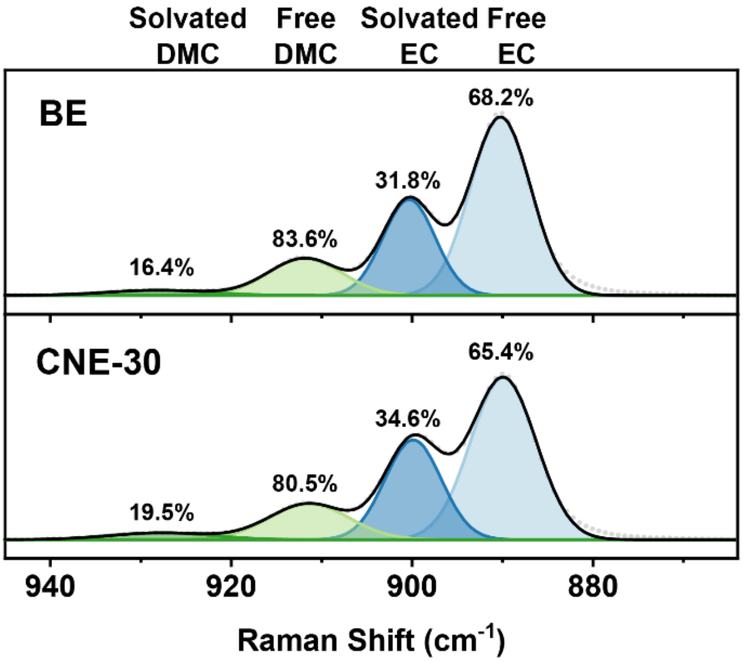

Fig. S3 High-resolution Raman spectra of the BE and the CNE-30 electrolyte in the range of 864-945 cm^{-1}

Notes: The peaks at 890, 900, 912, and 927 cm^{-1} correspond to the free EC, solvated EC, free DMC, and solvated DMC, respectively. Besides, the evolution of solvent in the Li$^+$-solvation structure was also explored through fitting the characteristic peaks in the Raman spectra, demonstrating the increased proportion of solvated EC and DMC in the CNE-30 electrolyte.

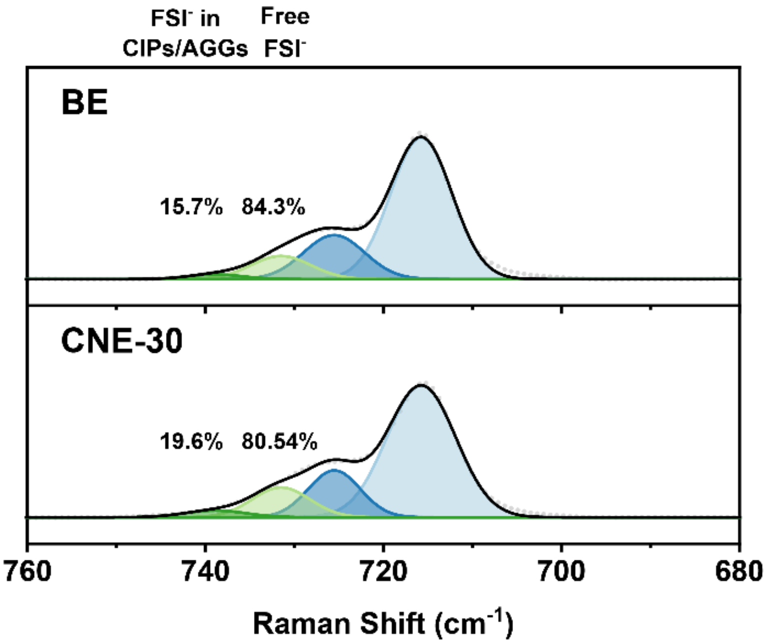

Fig. S4 High-resolution Raman spectra of the BE and the CNE-30 electrolyte in the range of 680-760 cm^{-1}

Notes: The peaks at 732 and 739 cm^{-1} correspond to the free FSI^- and FSI^- in CIPs/AGGs. Besides, the evolution of FSI^- in the Li^+-solvation structure was also explored through fitting the characteristic peaks in the Raman spectra, indicating the increased proportion of FSI^- in the CIPs/AGGs state.

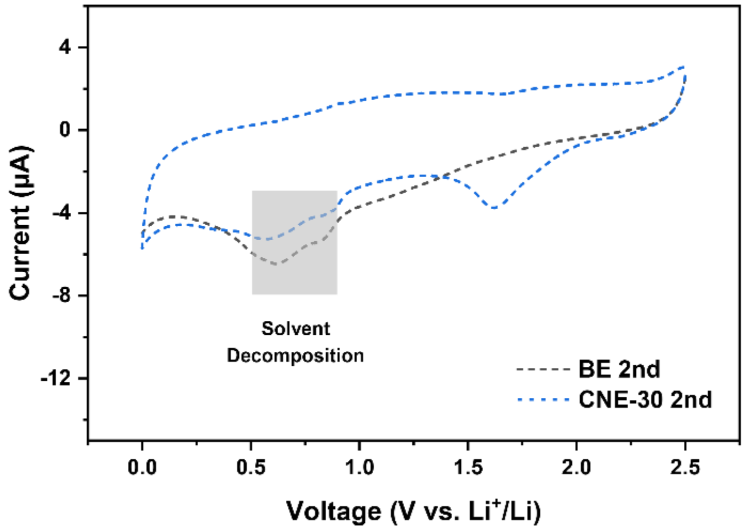

Fig. S5 CV curves of Li-Cu cells scanned at 0.1 mV s^{-1} in the voltage range of 0-2.5 V vs. Li^+/Li

Notes: The broad peak between 0.5 to 1.0 V is attributed to the solvent reduction which is greatly suppressed in the CNE-30 electrolyte during the 2nd cycle of CV. It is because as-formed SEI in the CNE-30 electrolyte is mainly from the preferential reduction of anions, mitigating the further reduction of electrolyte.

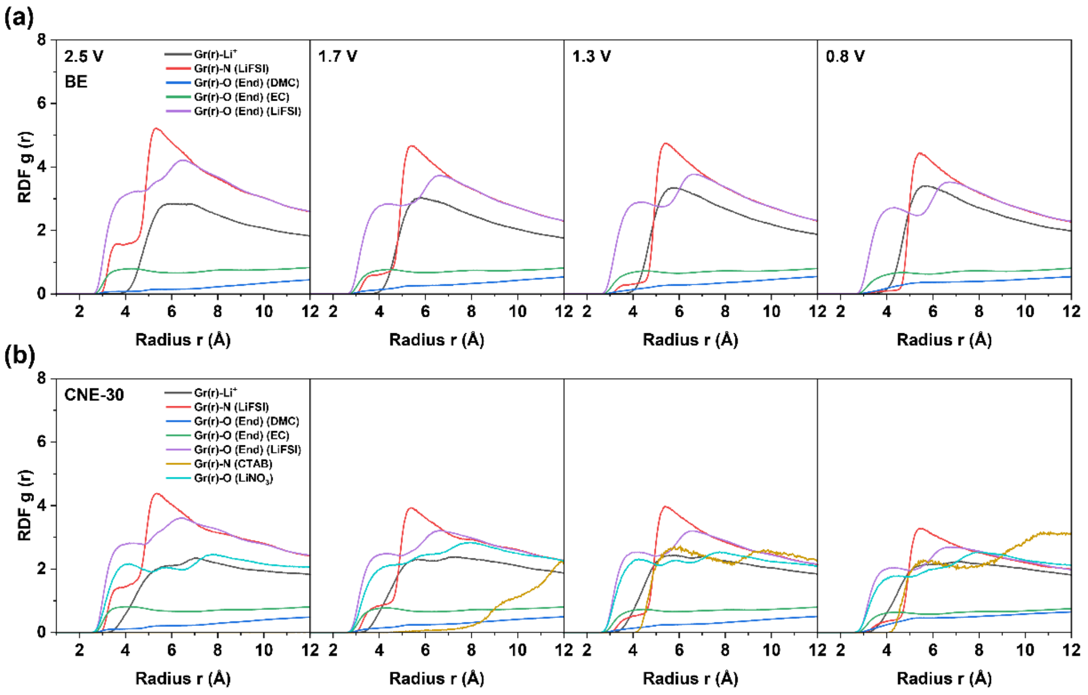

Fig. S6 Intensity profiles in (**a**) the BE and (**b**) the CNE-30 electrolyte as a function of distance (Å) from graphene electrode under different applied voltages (V)

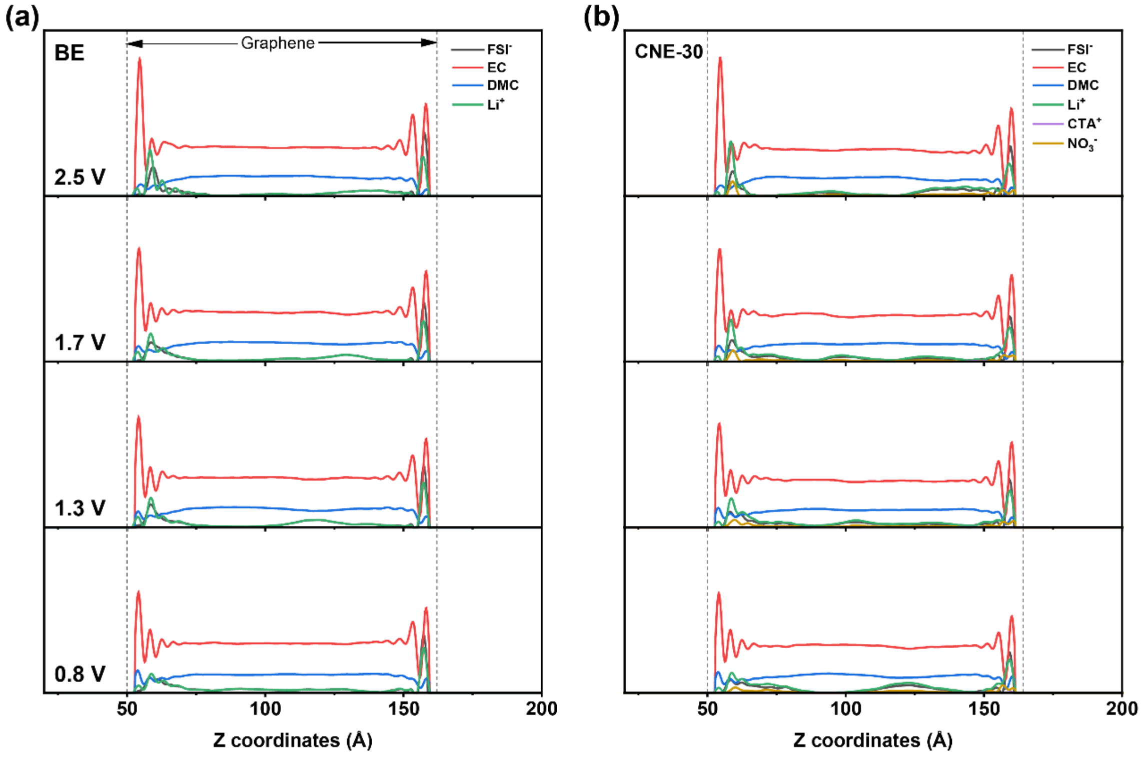

Fig. S7 Intensities of anions, cations, and solvent molecules in (**a**) the BE and (**b**) the CNE-30 electrolyte as a function of Z coordinates (Å) under different applied voltages (V). The potential of the left graphene electrode (as shown by short dash) is higher than the right one

Notes: The thickness of Inner Helmholtz plane (IHP) is determined to be 5.8 Å according to a peak position of EC solvent nearest to the graphene electrode on the right side, which is consistent with the consensus that the IHP is thinner than 1 nm within EDL region. Also, it can be seen in the figure that the thickness of IHP remains almost the same during different applied voltages.

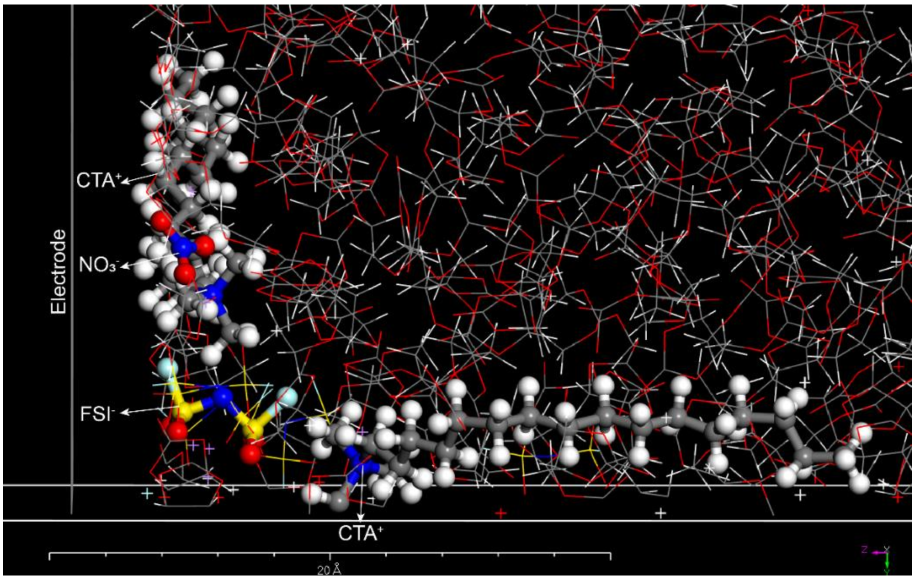

Fig. S8 Snapshots of EDL regions of the negative electrode in the CNE-30 electrolyte at 1.7 V

Notes: The selected clusters are shown in ball-stick structure and other solvent and ions are shown in line structure. In cluster 1 (CTA^+-NO_3^-), both NO_3^- and CTA^+ appear in the IHP. Similarly, in cluster 2 (CTA^+-FSI^-), FSI- also exists in the IHP with CTA^+ staying at the outer boundary of IHP.

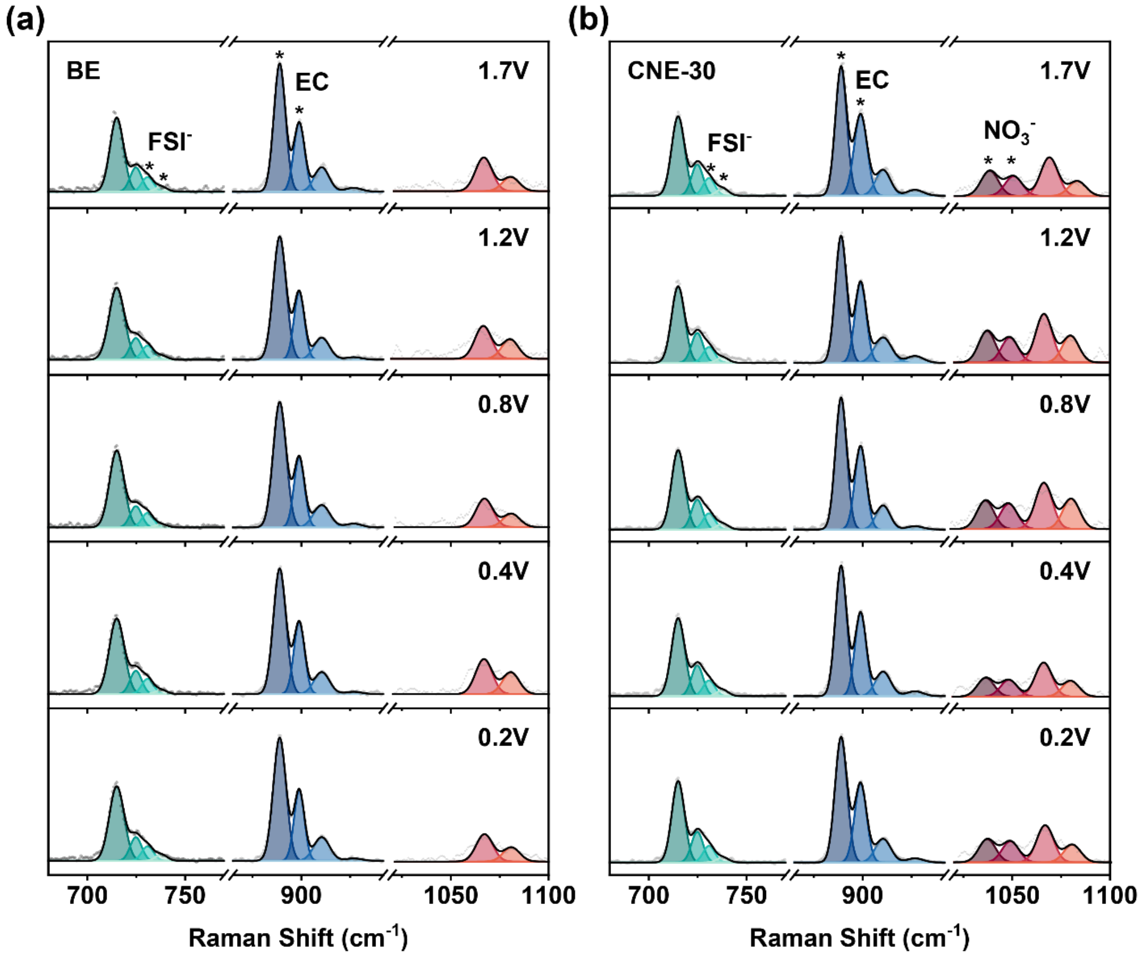

Fig. S9 Representative Raman spectra and peaking fitting under different voltages in (**a**) the BE and (**b**) the CNE-30 electrolyte

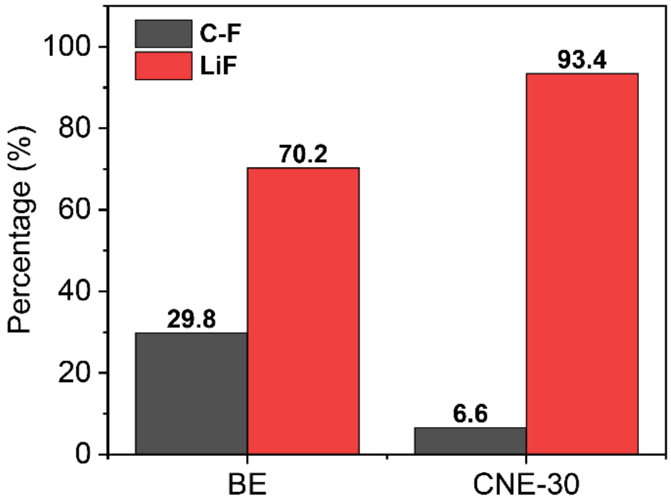

Fig. S10 Percentage of C-F and LiF species in the BE and the CNE-30 electrolyte calculated from F 1s spectra

Notes: The content of C-F is 29.8% in the BE electrolyte and 6.6% in the CNE-30 electrolyte according to XPS peak fitting in the F 1s spectra before Ar^+ suppering. The lower content of C-F in the CNE-30 electrolyte indicates the complete decomposition of LiFSI into inorganic components on the surface of LMA.

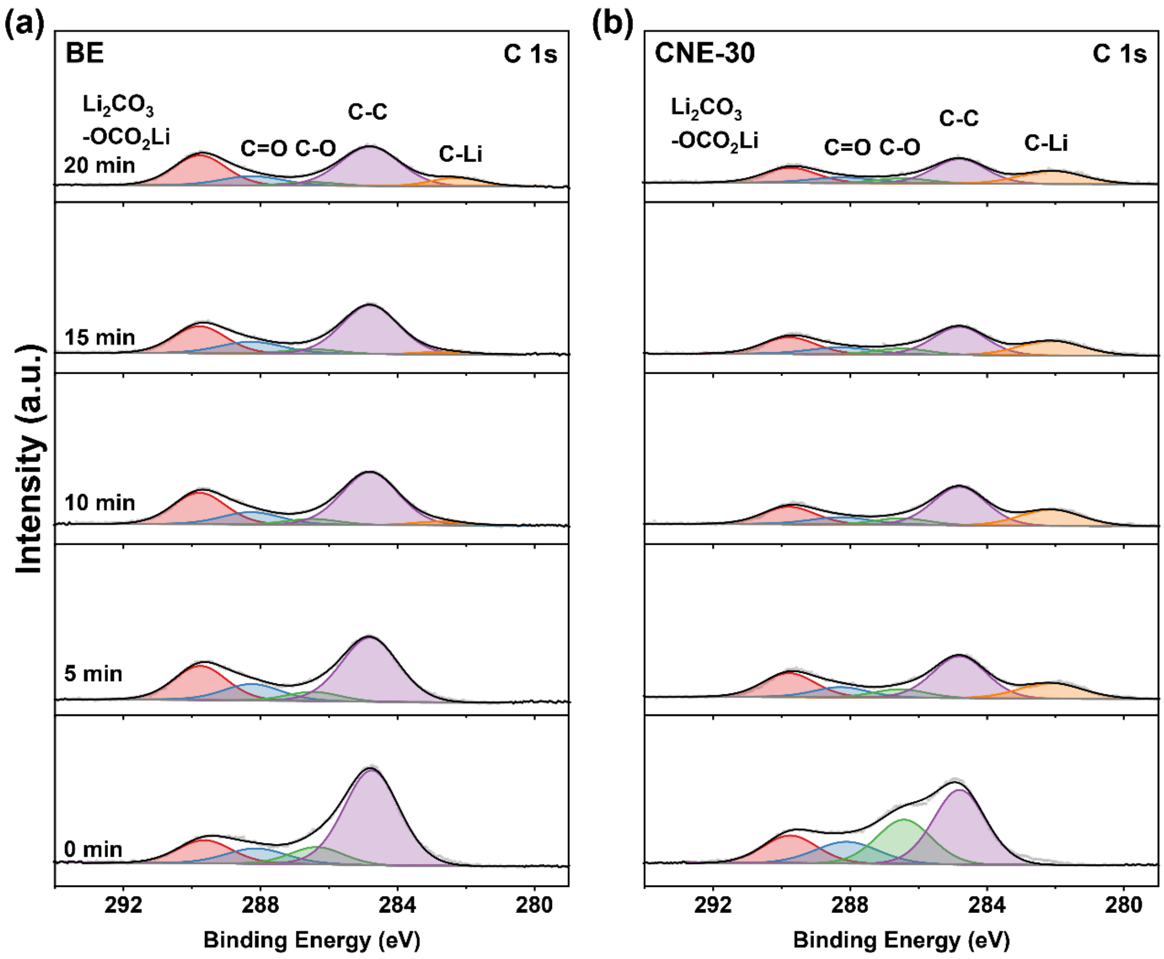

Fig. S11 XPS spectra of C 1s for SEI formed in (**a**) the BE and (**b**) the CNE-30 electrolyte and corresponding fitted curves

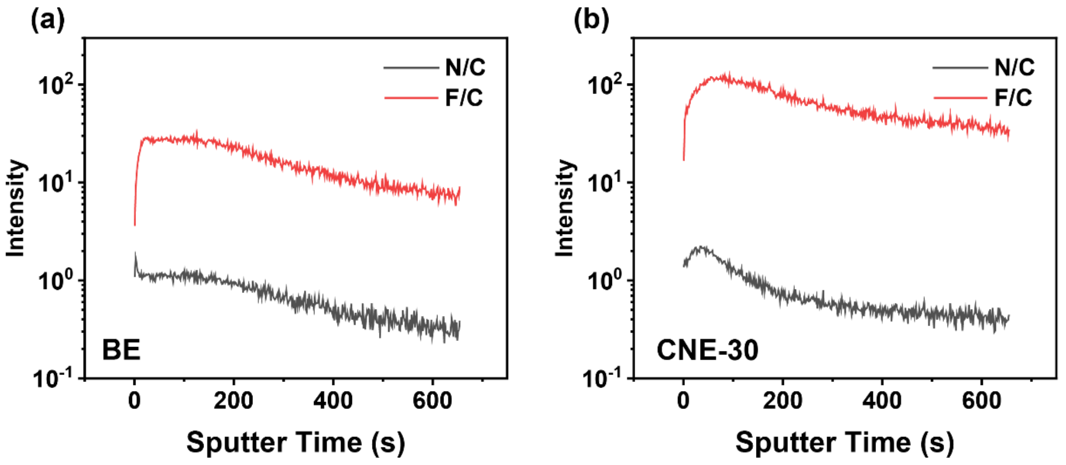

Fig. S12 ToF-SIMS spectra of N/C, F/C in (**a**) the BE and (**b**) the CNE-30 electrolyte

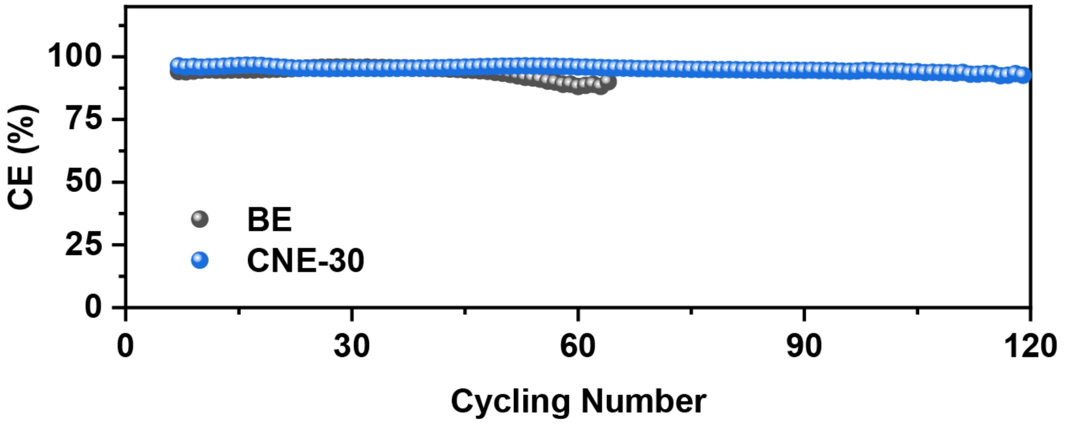

Fig. S13 Long-term cycling of Li-Cu half cells under 0.5 mA cm^{-2}/1 mAh cm^{-2}

Notes: As indicated in the figure, the CE of the BE electrolyte rapidly decays after the 50th. However, the CNE-30 can stably cycle 120 cycles with an average coulombic efficiency (ACE) value of 95.35% in the first 100 cycles.

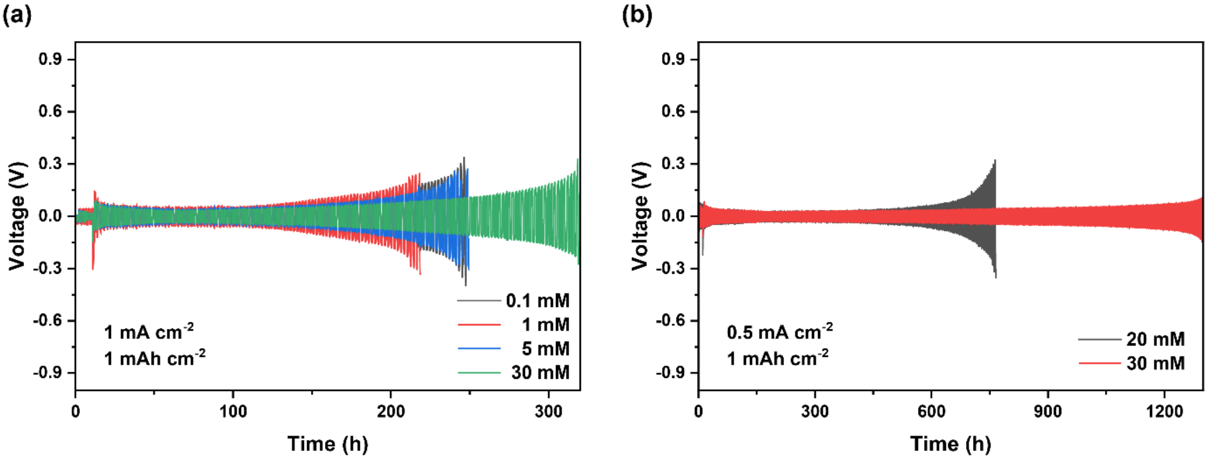

Fig. S14 Long-term cycling of Li-Li symmetric cells under (**a**) 0.5 mA cm^{-2}/1 mAh cm^{-2} and (**b**) 1 mA cm^{-2}/1 mAh cm^{-2} with various concentrations of CTAB

Notes: The best concentration of CTAB is determined based on two parameters: the ability of solubilizing $LiNO_3$ and cycling performance. First, 0.15 M $LiNO_3$ additive can be dissolved in the carbonate electrolyte (BE electrolyte) and no extra $LiNO_3$ can be solubilized with 0.1-5 mM CTAB. In the **Fig. S14a**, symmetric cells using carbonate electrolyte with 0.1-5 mM CTAB and

0.15 M $LiNO_3$ show shorter cycling lifetime (<250 h) than those with 30 mM CTAB and 0.3 M LiNO3 (320 h).

Second, in the **Fig. S14b**, though 20 mM CTAB can solubilize extra 0.05 M $LiNO_3$ (total 0.2 M $LiNO_3$) into the carbonate electrolyte, symmetric cells using carbonate electrolyte with 20 mM CTAB and 0.2 M $LiNO_3$ still show shorter cycling lifetime (<800 h) than those with 30 mM CTAB and 0.3 M $LiNO_3$ (1300 h). Due to the solubility of CTAB in the electrolyte being about 40mM, further increasing the concentration of CTAB does not result in additional dissolution of $LiNO_3$. As a summary, 30 mM CTAB is selected for the best choice for performance.

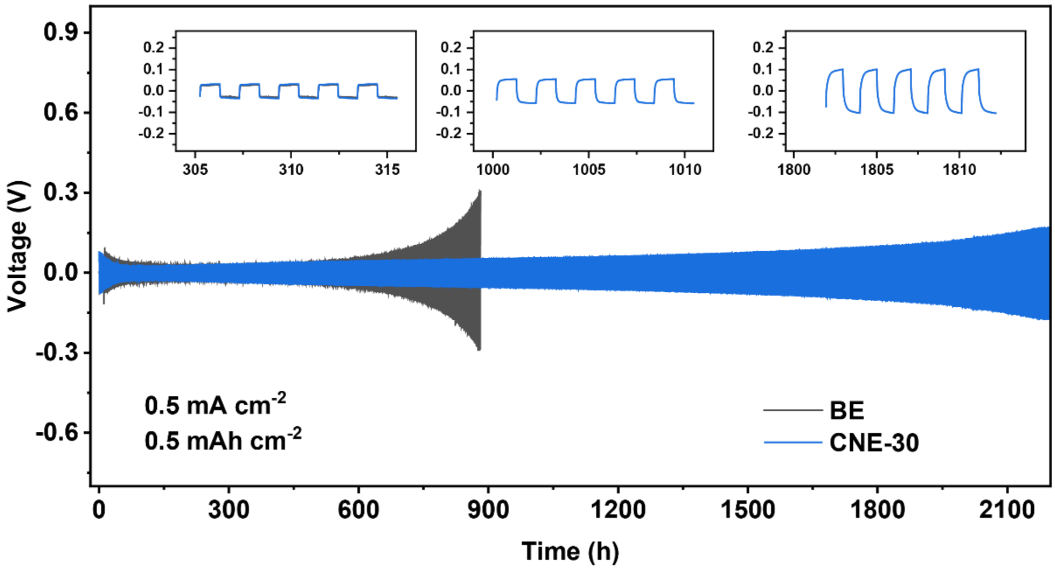

Fig. S15 Long-term cycling of Li||Li symmetric cells under 0.5 mA cm^{-2}/0.5 mAh cm^{-2} with insets showing magnified overpotential at 300 h/1000 h/1800 h respectively

Notes: Under a current density of 0.5 mA cm^{-2} with a capacity of 0.5 mAh cm^{-2}, the cycling time of the Li||Li cells with the CNE-30 electrolyte can be extended to more than 2200 h, while those with the BE electrolyte fail after 1200 h. It can be noted that the overpotential for Li||Li cells with the CNE-30 electrolyte remain as low as 50 mV around 1000 h.

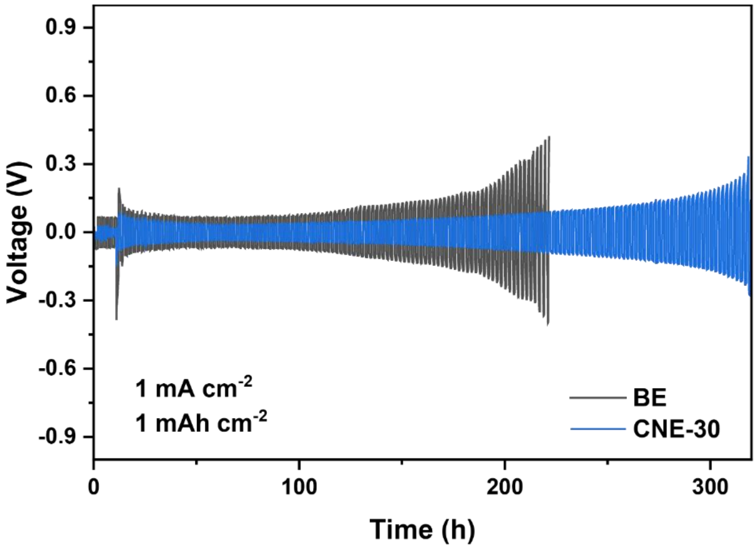

Fig. S16 Long-term cycling of Li-Li symmetric cells under 1 mA cm^{-2}/1 mAh cm^{-2} with the BE and CNE-30 electrolyte

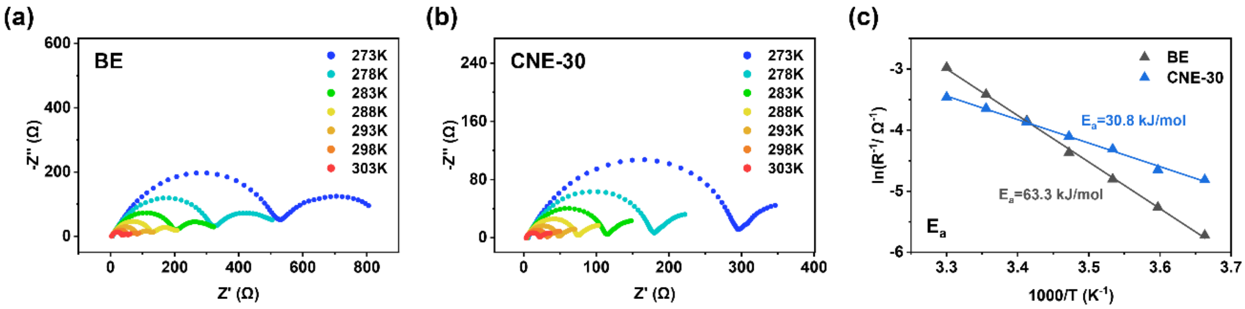

Fig. S17 EIS analysis of Li-Li cells under different temperatures (273 to 303 K) with (**a**) the BE and (**b**) the CNE-30 electrolyte; (**c**) corresponding activation energy calculated using Arrhenius equation

Notes: EIS analysis was performed on the Li||Li cells with the BE and the CNE-30 electrolyte under a series of temperatures ranging from 273 to 303 K, and then the first semi-circle at high frequency is fitted to obtain the R_{SEI}, which can be used to calculate the activation energy E_a.

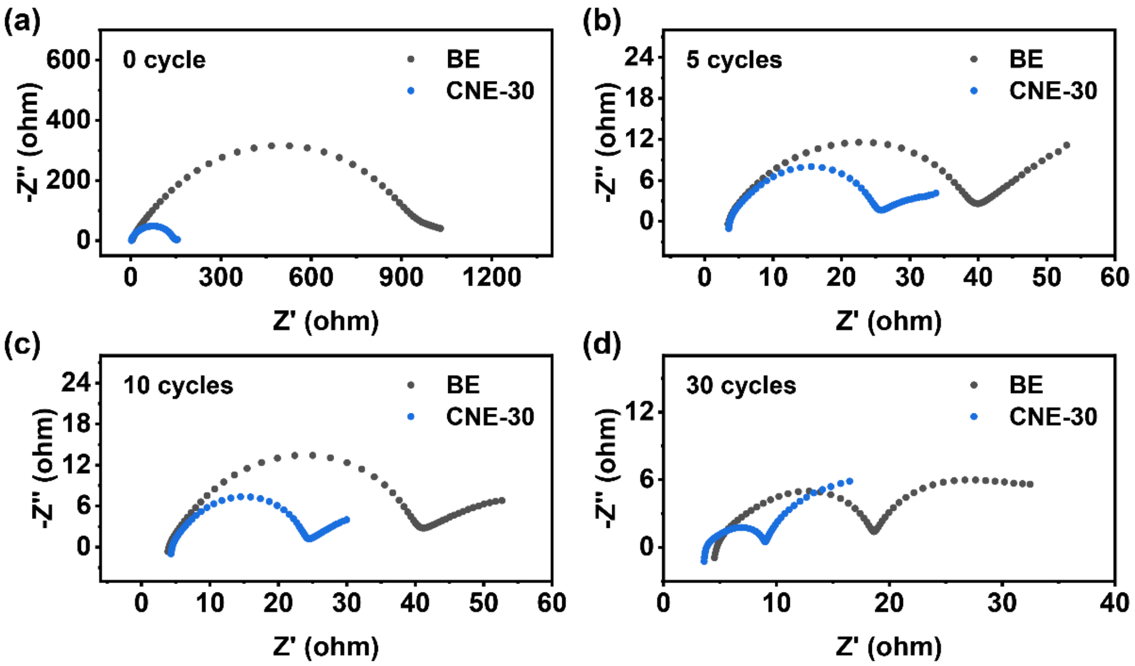

Fig. S18 EIS analysis of the BE and the CNE-30 electrolyte after (**a**) 0 cycle (**b**) 5 cycles (**c)** 10 cycles and (**d**) 30 cycles

Notes: Before cycling, the interfacial resistance of the cells in the CNE-30 (153.6 Ω) is much lower than that of the cells in the BE electrolyte (1010.2 Ω). Also, the interfacial resistance of the cells in both electrolytes decreases during cycling, with the cells in the CNE-30 electrolyte showing smaller resistance in all cycling conditions.

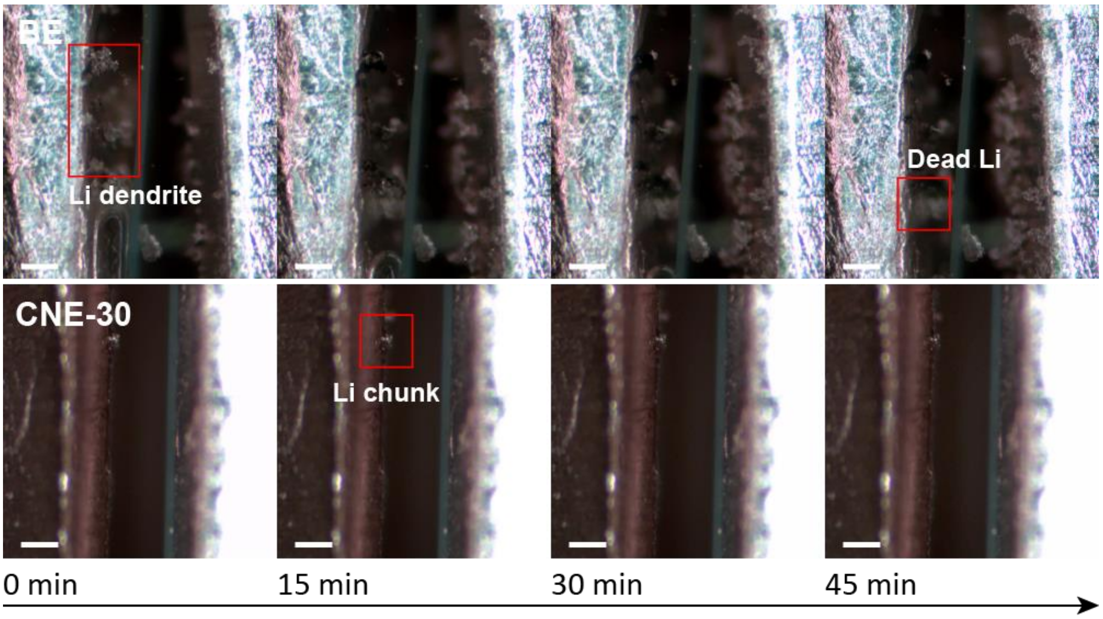

Fig. S19 *In-situ* optical images of Li stripping in the BE and the CNE-30 electrolyte (scale bars, 100 μm)

Notes: After 45 min of Li deposition on Li electrode, obvious dendrites can be observed in the BE electrolyte while chunky Li in the CNE-30 electrolyte. Then the deposited electrode was stripped under same current density (0.3 mA cm^{-2}). In the BE electrolyte, the root of Li dendrite is stripped away ahead of upper part of dendrite, resulting in dead Li in the electrolyte. At the same time, significant dendrites can be observed on the other electrode. In the contrast, in the CNE-30 electrolyte, deposited chunky Li is stripped away evenly, with no dead Li floating in the electrolyte.

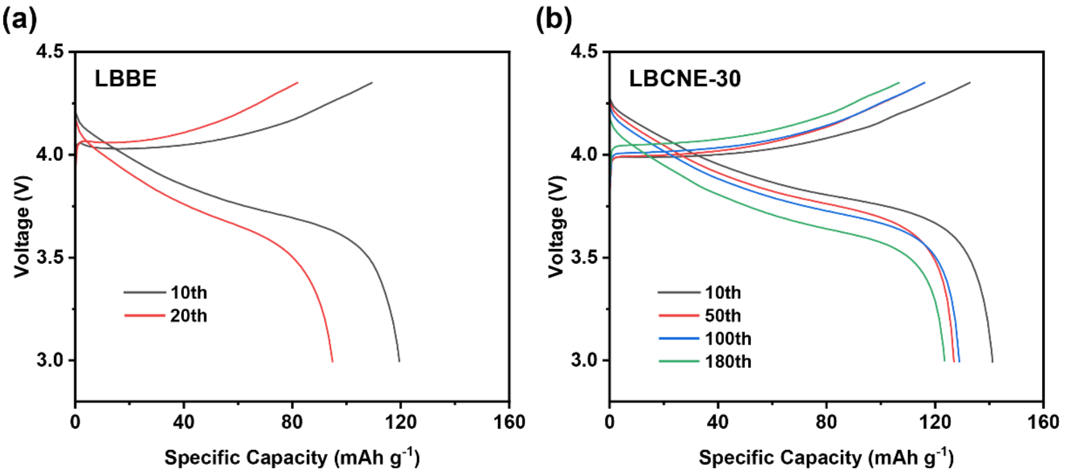

Fig. S20 Capacity-Voltage curves of Li||$LiCoO_2$ cells during the cycling with (**a**) the LBBE and (**b**) the LBCNE-30 electrolyte.

Notes: The Li||$LiCoO_2$ cell with the LBCNE-30 electrolyte shows better cycling stability compared to that with the LBBE electrolyte. From the discharge curve, we can see the full cell with the LBBE electrolyte undergoes more than 20% capacity loss while that with the LBCNE-30 electrolyte loses neglectable capacity (2.7%) from the 50th to 180th cycle.

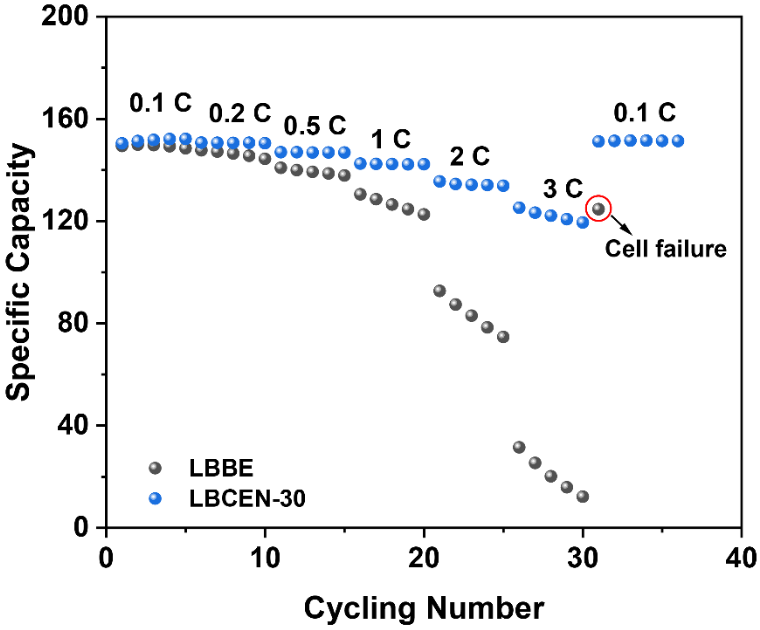

Fig. S21 Rate performance of the Li||$LiCoO_2$ full cells under 0.1/0.2/0.51/2/3 C using the LBBE and LBCNE-30 electrolyte

Notes: The Li||$LiCoO_2$ full cells using the LBBE and LBCNE-30 electrolyte can run rate test under higher rates of 0.1/0.2/0.5/1/2/3 C. In the **Fig. S21**, full cells with the LBBE electrolyte exhibit fast decay under 2 C and sudden cell failure when the rate is back to 0.1 C, showing inferior rate performance. However, the full cells with the LBCNE-30 electrolyte show capacities of 152/150/147/142/134/122 mAh g^{-1} under 0.1/0.2/0.5/1/2/3 C, respectively. Moreover, the full cells stably display a high capacity when the current density gets back to 0.1 C.

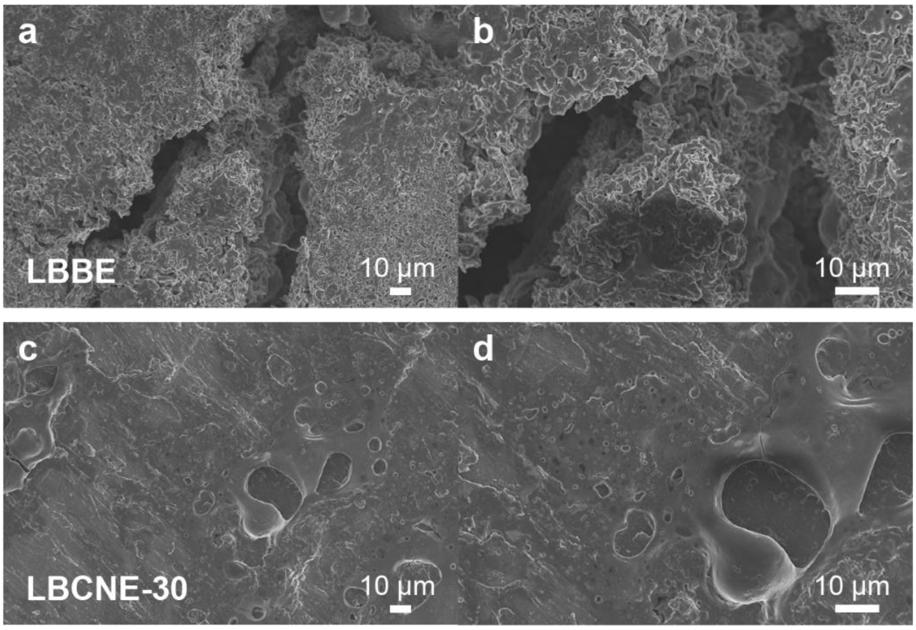

Fig. S22 SEM images of Li anode morphology after long-term cycling in Li||$LiCoO_2$ full cells in the (**a-b**) LBBE and (**c-d**) LBCNE-30 electrolyte

Notes: In the Fig. **S22a-b**, small-scale and needle-like lithium depositions can also be observed. Also, LMA with the LBBE electrolyte exhibits cracks on surface after cell failure which can be attributed to the accumulation of internal stress during cycling. The loosely compacted Li deposition further exacerbates volume expansion/shrinkage, rendering the surface more

susceptible to cracking. In the contrast, the Li depositions are relatively densely packed without visible Li dendrite growth in the LBCNE-30 electrolyte. Though some porous depositions appear on LMA using the LBCNE-30 electrolyte (**Fig.S22c-d**), no obvious cracks are on LMA. This is due to the inorganic-rich SEI of high mechanical strength, preventing cracks generation. Therefore, the cracks on LMAs have great influence on the fast decay of Li||$LiCoO_2$ full cells using the LBBE electrolyte.

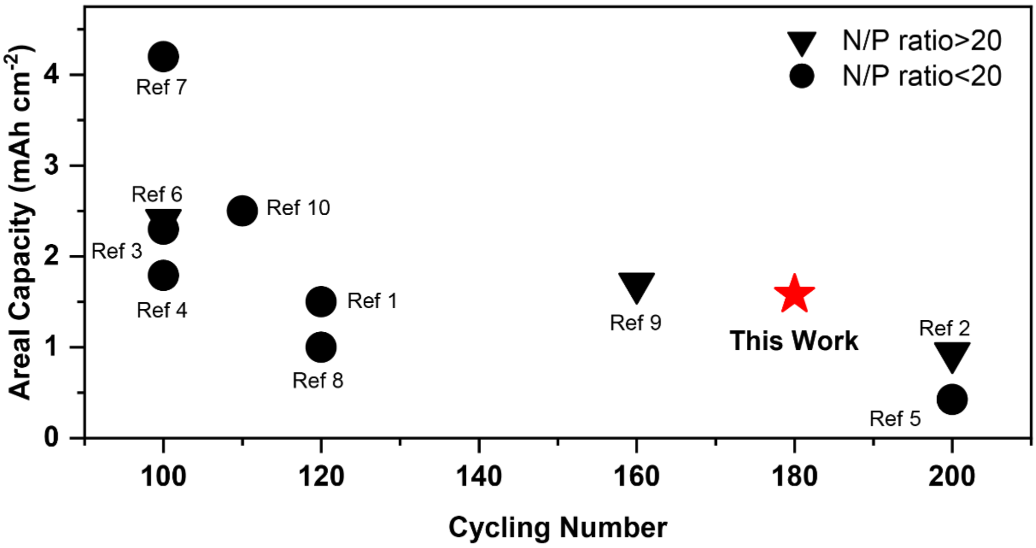

Fig. S23 Cycling performance comparison of Li|$LiFePO_4$ full cells in recent advanced electrolyte systems [S1–S10]

Table S1 Cycling time comparison of Li||Li symmetric cells in recently advanced electrolyte systems

**Electrolytes**	**Current density (mA cm^{-2})**	**Capacity (mAh cm^{-2})**	**Cycling time (h)**	**References**
1M LiFSI, 0.3 M $LiNO_3$, 30 mM CTAB in EC/DMC (7:3 by volume)	0.5<br>0.5	1<br>0.5	1300<br>2200	This work
0.7 M LiTFSI and 0.3 M $LiNO_3$ in EC/EGD (1:1 by volume)	0.5	0.5	800	[S11]
1 M LiFSI and 0.3 M $LiNO_3$ in FEC/GBL (1:2 by volume)	0.5	1	800	[S12]
1 M LiTFA in DME/FEC (7:3 by volume)	2.0	1	300	[S1]
1 M $LiPF_6$ in EC/DEC (1:1 by volume) with 0.2 wt% CuF_2 and 1 wt% $LiNO_3$	1	0.5	120	[S13]
1 M $LiPF_6$ in EC/DMC (1:1 by volume) with 5 wt% SL/$LiNO_3$ and 5 wt% FEC	0.5	0.5	750	[S14]
1 M $LiPF_6$ in EC/DMC/EMC (1:1:1 by volume) 25% $LiNO_3$ in Li anode	1	1	600	[S15]
1 M $LiPF_6$ in EC/DMC/TEGDME (2.4:5.6:2 by weight) with 3 wt% $LiNO_3$	1	1	800	[S16]
1 M $LiPF_6$ in FEC/EMC (3:7 by volume) with 1 wt% TPFPB and 3 wt% $LiNO_3$	1	1	700	[S17]
1 M $LiPF_6$ in EC/EMC (1:1 by volume) $LiNO_3$-rich layer @ Li	1	1	550	[S18]
1 M $LiPF_6$ in EC/DMC (1:1 by volume) with 2 wt% FEC, 1.6 wt% $LiNO_3$, 13.4 wt% TMU	1	1	350	[S19]
1 M $LiPF_6$ in EC/DMC (1:1 by volume) with 2 wt% FEC, 1.5 wt% $LiNO_3$, 12.3 wt% TPPO	1	1	1100	[S2]
1 M $LiPF_6$ in EC/DMC/EMC (1:1:1 by volume) with 1.2 wt% $LiNO_3$ and 3.6 wt% pyridine	1	1	300	[S20]
1 M $LiPF_6$ and 0.15 M TEAN in EC/DEC (1:1 by volume) with 10% FEC	1	1	500	[S3]
1 M LiTFSI in EC/DEC (1:1 by volume) with 5 vol% FEC and $Zn(TFSI)_2$ treatment	0.5	1	800	[S10]

Table S2 Corresponding values of R_{SEI} under different temperatures for the BE and the CNE-30 electrolyte

**T (K)**	**R_{SEI} (Ω) BE**	**R_{SEI} (Ω) CNE-30**
273	537.5	292.9
278	322.9	175.0
283	204.3	109.1
288	128.8	70.1
293	82.2	43.6
298	52.5	28.1
303	34.3	18.1

Table S3 Corresponding values of R_{SEI} after different cycling for the BE and the CNE-30 electrolyte

**Cycling Number**	**R_{SEI} (Ω) BE**	**R_{SEI} (Ω) CNE-30**
0	1010.2	153.6
5	41.1	22.9
10	37.5	20.0
30	13.9	6.2

Supplementary References

[S1] Z. Wang, F. Qi, L. Yin, Y. Shi, C. Sun, et al., An Anion-Tuned Solid Electrolyte Interphase with Fast Ion Transfer Kinetics for Stable Lithium Anodes. Adv. Energy Mater. **10**, 1903843 (2020). https://doi.org/10.1002/aenm.201903843

[S2] P. Xiao, R. Luo, Z. Piao, C. Li, J. Wang, et al., High-Performance Lithium Metal Batteries with a Wide Operating Temperature Range in Carbonate Electrolyte by Manipulating Interfacial Chemistry. ACS Energy Lett. **6**, 3170–3179 (2021). https://doi.org/10.1021/acsenergylett.1c01528

[S3] Z. Guo, X. Song, Q. Zhang, N. Zhan, Z. Hou, et al., Cationic Size Effect Promoting Dissolution of Nitrate Anion in Ester Electrolyte for Lithium–Metal Batteries. ACS Energy Lett. **7**, 569–576 (2022). https://doi.org/10.1021/acsenergylett.1c02495

[S4] T. Chen, J. You, R. Li, H. Li, Y. Wang, et al., Ultra-Low Concentration Electrolyte Enabling LiF-Rich SEI and Dense Plating/Stripping Processes for Lithium Metal Batteries. Adv. Sci. **9**, 2203216 (2022). https://doi.org/10.1002/advs.202203216

[S5] T. Zhou, Y. Zhao, M. El Kazzi, J. W. Choi, and A. Coskun, Stable Solid Electrolyte Interphase Formation Induced by Monoquat-Based Anchoring in Lithium Metal Batteries. ACS Energy Lett. **6**, 1711–1718 (2021). https://doi.org/10.1021/acsenergylett.1c00274

[S6] L. Xiao, X. Chen, R. Cao, J. Qian, H. Xiang, et al., Enhanced Performance of Li|LiFePO4 Cells Using CsPF6 as an Electrolyte Additive. J. Power Sources **293**, 1062–1067 (2015). https://doi.org/10.1016/j.jpowsour.2015.06.044

[S7] D. Wang, H. Liu, M. Li, D. Xia, J. Holoubek, et al., A Long-Lasting Dual-Function Electrolyte Additive for Stable Lithium Metal Batteries. Nano Energy **75**, 104889 (2020). https://doi.org/10.1016/j.nanoen.2020.104889

[S8] S. Kim, K. Cho, J. Kwon, K. Sim, D. Seok, et al., An Inorganic-Rich SEI Layer by the Catalyzed Reduction of $LiNO_3$ Enabled by Surface-Abundant Hydrogen Bonding for

Stable Lithium Metal Batteries. Small **19**, 2207222 (2023). https://doi.org/10.1002/smll.202207222

[S9] D. Zhang, R. Gu, W. Guo, Q. Xu, H. Li, et al., Long-Life and High-Rate-Charging Lithium Metal Batteries Enabled by a Flexible Active Solid Electrolyte Interphase Layer. ACS Appl. Mater. Interfaces **13**, 60678–60688 (2021). https://doi.org/10.1021/acsami.1c19952

[S10] J. Chen, Z. Sun, Z. Li, J. Liu, X. Yao, et al., Highly Reversible Li Metal Anode Using a Binary Alloy Interface. Chem. Commun. **58**, 13455–13458 (2022). https://doi.org/10.1039/D2CC05051J

[S11] H. Yang, X. Chen, N. Yao, N. Piao, Z. Wang, et al., Dissolution–Precipitation Dynamics in Ester Electrolyte for High-Stability Lithium Metal Batteries. ACS Energy Lett. **6**, 1413–1421 (2021). https://doi.org/10.1021/acsenergylett.1c00149

[S12] Y. Jie, X. Liu, Z. Lei, S. Wang, Y. Chen, et al., Enabling High-Voltage Lithium Metal Batteries by Manipulating Solvation Structure in Ester Electrolyte. Angew. Chem. Int. Ed. **59**, 3505–3510 (2020). https://doi.org/10.1002/anie.201914250

[S13] C. Yan, Y.-X. Yao, X. Chen, X.-B. Cheng, X.-Q. Zhang, et al., Lithium Nitrate Solvation Chemistry in Carbonate Electrolyte Sustains High-Voltage Lithium Metal Batteries. Angew. Chem. Int. Ed. **57**, 14055–14059 (2018). https://doi.org/10.1002/anie.201807034

[S14] N. Piao, S. Liu, B. Zhang, X. Ji, X. Fan, et al., Lithium Metal Batteries Enabled by Synergetic Additives in Commercial Carbonate Electrolytes. ACS Energy Lett. **6**, 1839–1848 (2021). https://doi.org/10.1021/acsenergylett.1c00365

[S15] L. Fu, X. Wang, L. Wang, M. Wan, Y. Li, et al., A Salt-in-Metal Anode: Stabilizing the Solid Electrolyte Interphase to Enable Prolonged Battery Cycling. Adv. Funct. Mater. **31**, 2010602 (2021). https://doi.org/10.1002/adfm.202010602

[S16] D. Xiao, Q. Li, D. Luo, G. Li, H. Liu, et al., Regulating the Li^+-Solvation Structure of Ester Electrolyte for High-Energy-Density Lithium Metal Batteries. Small **16**, 2004688 (2020). https://doi.org/10.1002/smll.202004688

[S17] S. Li, W. Zhang, Q. Wu, L. Fan, X. Wang, et al., Synergistic Dual-Additive Electrolyte Enables Practical Lithium-Metal Batteries. Angew. Chem. Int. Ed. **59**, 14935–14941 (2020). https://doi.org/10.1002/anie.202004853

[S18] X. Wang, H. Wang, M. Liu, and W. Li, In-Plane Lithium Growth Enabled by Artificial Nitrate-Rich Layer: Fast Deposition Kinetics and Desolvation/Adsorption Mechanism. Small **16**, 2000769 (2020). https://doi.org/10.1002/smll.202000769

[S19] Z. Piao, P. Xiao, R. Luo, J. Ma, R. Gao, et al., Constructing a Stable Interface Layer by Tailoring Solvation Chemistry in Carbonate Electrolytes for High-Performance Lithium-Metal Batteries. Adv. Mater. **34**, 2108400 (2022). https://doi.org/10.1002/adma.202108400

[S20] D. Liu, X. Xiong, Q. Liang, X. Wu, and H. Fu, An Inorganic-Rich SEI Induced by $LiNO_3$ Additive for a Stable Lithium Metal Anode in Carbonate Electrolyte. Chem. Commun. **57**, 9232–9235 (2021). https://doi.org/10.1039/D1CC03676A
